# Reattachable fiducial skin marker for automatic multimodality registration

**DOI:** 10.1007/s11548-022-02639-7

**Published:** 2022-05-23

**Authors:** Benjamin J. Mittmann, Alexander Seitel, Gernot Echner, Wiebke Johnen, Regula Gnirs, Lena Maier-Hein, Alfred M. Franz

**Affiliations:** 1 Medical Faculty, Heidelberg University, Im Neuenheimer Feld 672, 69120 Heidelberg, BW Germany; 2 Department of Computer Science, Ulm University of Applied Sciences, Albert-Einstein-Allee 55, 89081 Ulm, BW Germany; 3 Department of Medical Physics in Radiation Oncology, German Cancer Research Center (DKFZ), Im Neuenheimer Feld 280, 69120 Heidelberg, BW Germany; 4 Department of Radiology, German Cancer Research Center (DKFZ), Im Neuenheimer Feld 280, 69120 Heidelberg, BW Germany; 5 Department of Computer Assisted Medical Interventions, German Cancer Research Center (DKFZ), Im Neuenheimer Feld 223, 69120 Heidelberg, BW Germany; 6 Faculty of Mathematics and Computer Science, Heidelberg University, Im Neuenheimer Feld 205, 69120 Heidelberg, BW Germany

**Keywords:** Reattachable fiducial skin marker, Automatic multimodality registration, Fiducial marker assessment

## Abstract

**Purpose:**

Fusing image information has become increasingly important for optimal diagnosis and treatment of the patient. Despite intensive research towards markerless registration approaches, fiducial marker-based methods remain the default choice for a wide range of applications in clinical practice. However, as especially non-invasive markers cannot be positioned reproducibly in the same pose on the patient, pre-interventional imaging has to be performed immediately before the intervention for fiducial marker-based registrations.

**Methods:**

We propose a new non-invasive, reattachable fiducial skin marker concept for multi-modal registration approaches including the use of electromagnetic or optical tracking technologies. We furthermore describe a robust, automatic fiducial marker localization algorithm for computed tomography (CT) and magnetic resonance imaging (MRI) images. Localization of the new fiducial marker has been assessed for different marker configurations using both CT and MRI. Furthermore, we applied the marker in an abdominal phantom study. For this, we attached the marker at three poses to the phantom, registered ten segmented targets of the phantom’s CT image to live ultrasound images and determined the target registration error (TRE) for each target and each marker pose.

**Results:**

Reattachment of the marker was possible with a mean precision of 0.02 mm ± 0.01 mm. Our algorithm successfully localized the marker automatically in all ($n=201$) evaluated CT/MRI images. Depending on the marker pose, the mean ($n=10$) TRE of the abdominal phantom study ranged from 1.51 ± 0.75 mm to 4.65 ± 1.22 mm.

**Conclusions:**

The non-invasive, reattachable skin marker concept allows reproducible positioning of the marker and automatic localization in different imaging modalities. The low TREs indicate the potential applicability of the marker concept for clinical interventions, such as the puncture of abdominal lesions, where current image-based registration approaches still lack robustness and existing marker-based methods are often impractical.

**Supplementary Information:**

The online version contains supplementary material available at 10.1007/s11548-022-02639-7.

## Introduction

With the wealth of imaging modalities available, fusing image information has become increasingly important for optimal diagnosis and treatment of the patient. For this purpose, the images are registered, i.e. they are transformed to a common coordinate system. Among others, *intensity-based* [1, 6, 21] and *geometry-based* [2, 9, 13, 22] approaches have become the gold standard for this task in the last decades. These either use voxel and pixel values to calculate similarity measures of both images (*intensity-based*) [9] or identify corresponding, geometric image features, such as anatomical landmarks, artificial points or edges for image alignment (*geometry-based*) [14]. Intensity-based registrations require no additional hardware, but they are computationally more expensive and less robust in their application compared to geometry-based registrations [21]. Recently, however, *deep learning-based* methods [10, 15, 25] using both image intensities and geometric image features gained more and more attraction with an increasing number of promising approaches published in the last three years [10].

Often, so-called *fiducial markers* are applied to provide artificial, geometric image features. They contain artificial structures, the *fiducial features*, which are well localizable in the images of both imaging modalities. Fiducial markers are either surgically implanted or non-invasively attached on the patient’s skin as so-called *skin markers*. By introducing those artificial markers, the registration methods can in particular be optimized regarding the robustness and accuracy in feature detection and thus the registration accuracy itself [18, 24]. Additionally, they enable to perform registrations with commonly used tracking modalities of image guidance systems. The ACUSTAR system, e.g., was one of the first image guidance systems proposing the use of an implantable, reattachable fiducial marker [7, 8, 18]. However, tissue inflammations around the implanted marker may occur. Another reattachable, invasively usable fiducial marker concept was proposed by *van Beek et al.* in 2017 [23].

Furthermore, relatively small computed tomography (CT) and magnetic resonance imaging (MRI) fiducial skin markers were developed by several companies. Probably, these could remain on the patient for several hours, but once they are detached from the patient, reattaching them exactly in the same pose is possibly not feasible. Hence, common fiducial skin markers must not be removed from and reattached on the patient during the registration process, and almost none of the existing skin markers allow a time gap of 1 or 2 days between pre- and intra-interventional imaging.

Localizing the fiducial marker in the image was conducted automatically [5, 22, 26], semi-automatically [3, 16, 20] or manually [12, 13]. However, fully automatic marker localization remains challenging with respect to different imaging modalities, various fiducial marker configurations and algorithm robustness. In summary, existing fiducial markers lead to restrictions in clinical practice. They lack easy and fast applicability, reattachability and robust automatic localization approaches.

**Fig. 1:**
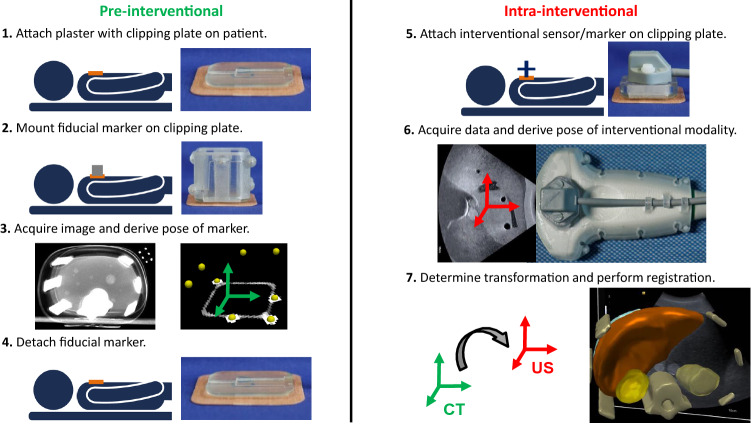
Proposed workflow of a non-invasive, reattachable fiducial skin marker applicable for multi-modal interventional registration approaches

We propose a new reattachable fiducial skin marker concept that was designed for multi-modal interventional registration approaches including the use of electromagnetic (EM) or optical tracking technologies (Fig. 1). As a use case, we focus on minimally-invasive abdominal interventions and explicitly address the specific requirements of these interventions by the chosen marker design. These could benefit from the non-invasive marker application and its ability to perform pre-interventional imaging one or two days before the planned intervention. We furthermore describe a robust, automatic localization algorithm for CT/MRI images, thoroughly evaluate individual parts of the concept prototype and use it to register CT images to live ultrasound (US) images.

## Methods

In the following sections, the marker design details (section “Reattachable fiducial skin marker”), the marker localization (section “Fiducial marker localization in CT/MRI images”), the marker assessments (section “Fiducial marker assessments”) and the marker application in an abdominal phantom study (section “Abdominal phantom study: use case CT-to-US registration”) are described.

### Reattachable fiducial skin marker

**Fig. 2:**
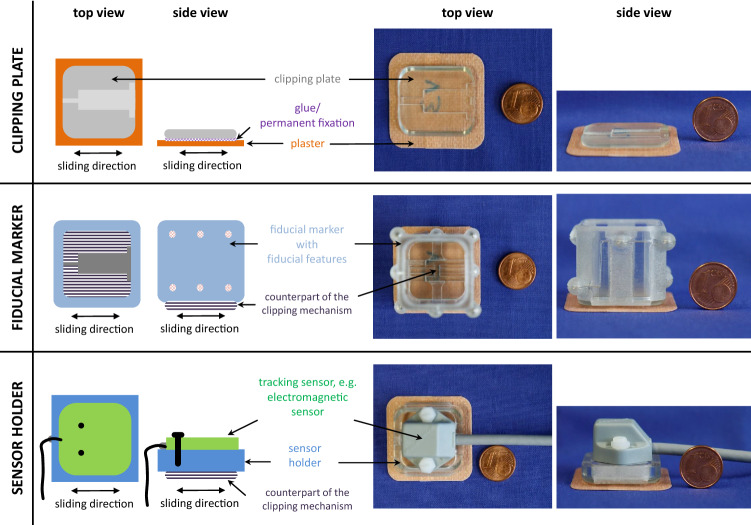
Proposed reattachable fiducial skin marker concept consisting of a clipping plate (component 1), a fiducial marker (component 2) and a sensor holder (component 3). The fiducial marker and the sensor holder are reproducibly (re)attachable on the clipping plate

The skin marker concept consists of three components (Fig. 2): a marker platform, referred to as *clipping plate*, a *fiducial marker* and a *sensor holder*. These are applicable in multi-modal registration workflows of image guided interventions (Fig. 1).

***Clipping plate*** A conventional plaster serves as fixation aid to attach the small and thin clipping plate on the patient’s skin. The plate provides a clipping mechanism for mounting the fiducial marker or the sensor holder on the clipping plate. When mounted on the clipping plate, the fiducial marker and the sensor holder have a known, constant, pose relative to each other.

***Fiducial marker*** The fiducial marker contains eight exchangeable, spherical fiducial features, which were uniquely distributed over two parallel planes with four fiducial features in each plane. The fiducial marker can be adapted to different imaging modalities, such as CT or MRI.

***Sensor holder*** The sensor holder allows to receive sensors of different tracking systems in a geometrically predefined pose. For our abdominal phantom study described below, we configured it to be usable with the EM *RX2* sensor (Polhemus Inc., Colchester, VT, USA).

Further design details and a workflow description of using the marker concept for multimodality registrations in image guidance systems is given in *Online Resource 1*.

### Fiducial marker localization in CT/MRI images

Basic image filtering steps and the prior knowledge of the fiducial features’ distance configuration are used for an automatic fiducial marker localization for both CT and MRI images.

**Fig. 3:**
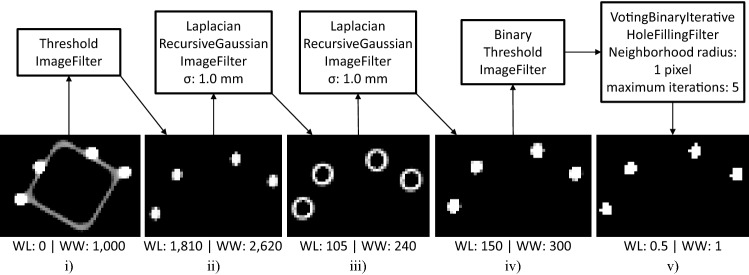
ITK filter pipeline for identifying fiducial feature candidates and illustration of the filter outputs when using a CT image of the fiducial marker. A volume rendered view of the corresponding whole CT scan is given in Fig. 6b. Finally, contiguous white voxel regions are treated as fiducial feature candidates. *WL* window level, *WW* window width

Detecting fiducial feature candidates within an imaging volume is based on an Insight Toolkit (ITK [19]) filter pipeline (Fig. 3) that is configured to be self-adjustable depending on the image input. It filters out negligible soft tissue signals (Fig. 3i–ii), localizes the fiducial features based on their characterized large voxel value gradients of their edge regions (Fig. 3ii–iv), and binarizes the image for being able to identify fiducial feature candidates (Fig. 3v) in a label map using the ITK *BinaryImageToShapeLabelMapFilter*. The candidate list is then pruned by excluding candidates with a volume smaller than 0.6 times (CT${\mid }$MRI) or greater than two times (MRI only) the real fiducial feature volume. As the eight fiducial features of the fiducial marker (component 2, Fig. 2) have a unique geometric distance configuration to each other, candidates for which no known distance configuration applies are iteratively excluded. The remaining fiducial feature candidates are then ordered according to the geometrically known fiducial feature distribution (see *Online Resource 1*) and the pose of the fiducial marker is determined by using the point-based method proposed by *Horn* [11]. More detailed information about the detection pipeline is provided in *Online Resource 1*.

### Fiducial marker assessments

We investigated two main aspects for assessing the fiducial marker: the *clipping precision* provided by the clipping mechanism and the *localization error* of the marker itself (component 2, Fig. 2).

#### Clipping precision

To assess the *clipping precision*, we clipped the sensor holder with a rigidly attached passive, optically localizable sensor 40 times on and off the clipping plate and determined the pose of the sensor holder in its mounted state with respect to a simultaneously localized reference sensor, which was required to be able to compensate temporal measurement data drifts. Finally, we calculated L2-norm-based metrics with respect to the translational and the rotational deviations $\epsilon _{\mathrm{trans}}$ and $\epsilon _{\mathrm{rot}}$ between the reference sensor and the clipped-on sensor holder (Eqs. 1 and 2) and treated these as a measure of the *clipping precision*.

1 $$\begin{aligned} \epsilon _{\mathrm{trans}}= & {} \frac{1}{40} \sum \limits _{i=1}^{40} {\mid } l_i - l_{\mathrm{mean}} {\mid } \end{aligned}$$

2 $$\begin{aligned} \epsilon _{\mathrm{rot}}= & {} \frac{1}{40} \sum \limits _{i=1}^{40} {\mid } {\mid } \varvec{\theta _{i}} - \varvec{\theta _{\mathrm{mean}}} {\mid } {\mid } \end{aligned}$$

For determining $\epsilon _{\mathrm{trans}}$, we averaged the absolute values between $l_i$ and $l_{\mathrm{mean}}$, where $l_i$ denotes the *i*th L2-norm distance between the reference sensor and the sensor holder and $l_{\mathrm{mean}}$ denotes the average of $l_{1},{\ldots ,40}$. $\epsilon _{\mathrm{rot}}$ was determined by averaging the L2-norms between $\varvec{\theta _i}$ and $\varvec{\theta _{\mathrm{mean}}}$, where $\varvec{\theta _i}$ indicates the *i*th vector of the Euler angles describing the transformation from the pose of the reference sensor to the pose of the sensor holder, and $\varvec{\theta _{\mathrm{mean}}}$ denotes the mean vector of $\varvec{\theta _{1},{\ldots ,40}}$.

To investigate rigid and skin-mimicking mounting conditions, we placed the clipping plate at three different positions on the *Image-Guided Abdominal Biopsy Phantom, Model 071B* (Computerized Imaging Reference System, Inc., Norfolk, VA, USA), as illustrated in Fig. 6a, and determined $\epsilon _{\mathrm{trans}}$ and $\epsilon _{\mathrm{rot}}$ for each clipping position. Details regarding the experimental setup and the optical tracking system used in this experiment are provided in *Online Resource 2*.

#### Localization error

**Fig. 4:**
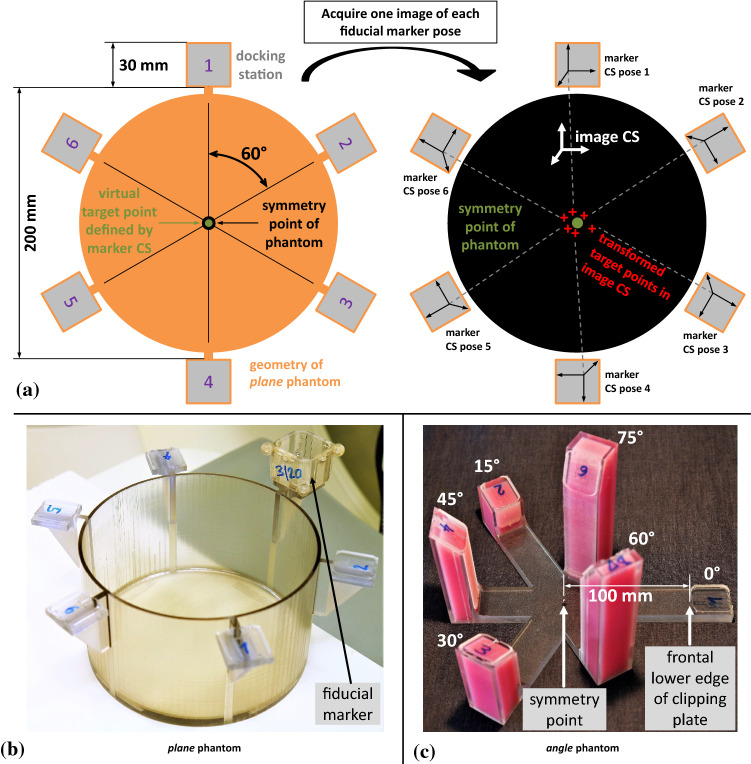
**a** Schematic approach to the use of the *plane* phantom. Note: The non-symmetric dashed lines in the right image indicate the deviations caused by the localization error. **b**, **c** Pictures of the *plane*/*angle* phantom. The relative Euclidean distance between the symmetry point and any docking station is 100 mm

For determining the localization error we present an experimental setup consisting of two measurement phantoms that makes use of a point symmetric placement of the marker in different poses (Fig. 4). This setup allows predicting the position of a virtual target and deriving a measure of fiducial marker localization that is not dependent on reference annotation variability due to inter-observer variability and image resolution. We refer to this measure as *point prediction error* (PPE). The details regarding the measurement phantoms, the assessment procedure as well as the assessments for two modalities (CT${\mid }$MRI) are explained in the following.

**Fig. 5:**
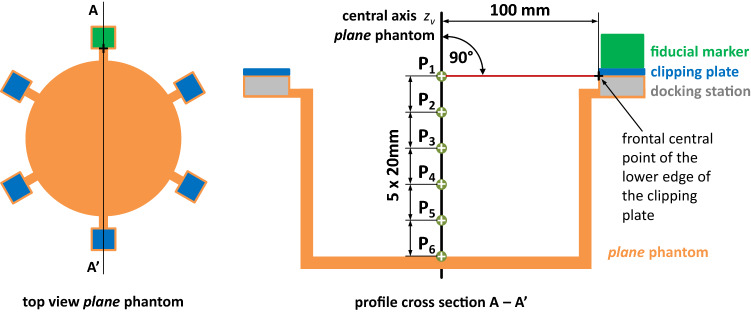
Positions of the six virtual target points $P_{1,\ldots ,6}$, which were considered in case of the *plane* phantom. In case of the *angle* phantom, only one virtual target point (target distance: 100 mm) was considered because of its angular geometry

***Assessment phantoms*** The two high precision measurement phantoms serve as a mechanical platform to investigate marker placement at predefined poses in the imaging volume. Each phantom provides six docking stations for the marker. The stations are evenly and point symmetrically arranged around the symmetry point of the phantom (Fig. 4a), and they allow planar (*plane* phantom) and angulated (*angle* phantom) marker placement in a volume roughly reflecting realistic intervention scenarios. The symmetry of the marker positions with respect to the phantom’s symmetry point allows calculating a prediction error, the PPE, of a virtual target solely based on the six individual marker poses. By using the *plane* phantom, the PPE can be determined with respect to multiple virtual targets (Fig. 5). The *angle* phantom is utilized to assess the influence of a non-planar, angulated placement of the marker on the PPE with respect to a constant target distance.

***Assessment procedure*** Assessment of the marker was accomplished as follows:

1. The fiducial marker was mounted consecutively to each docking station and one volume image was acquired of each marker pose.
2. The previously described localization algorithm (“Fiducial marker localization in CT/MRI images” section) was applied on each image resulting in the transformations $T_1,\ldots ,T_6$ from the marker coordinate system (CS) to the image CS.
3. The transformations $T_1,\ldots ,T_6$ were applied to the virtual target point(s), as defined in Fig. 5, resulting in a set of six transformed points $p_1,\ldots ,p_6$ for each virtual target point.
4. The PPE was calculated according to Eq. 3 for each virtual target point. Further details regarding the PPE, its meaning and its calculation are provided in *Online Resource 2*.
  3 $$\begin{aligned} \hbox {PPE} = \sqrt{\frac{\sum \nolimits _{i=1}^6(p_{i,x} - \bar{p}_x)^2 + (p_{i,y} - \bar{p}_y)^2 + (p_{i,z} -\bar{p}_z)^2}{6}} \nonumber \\ \end{aligned}$$

$p_i$    coordinate vector of the *i*th transformed point

$\bar{p}$    coordinate vector of the approximated symmetry point, i.e. mean ($p_1,\ldots ,p_6$)

$\bar{p}_x$    scalar x-coordinate of $\bar{p}$. $\bar{p}_y$ and $\bar{p}_z$ accordingly

***Assessment for CT/MRI imaging*** To quantitatively determine the impact of different marker design parameters on the localization error for the presented fiducial marker and to investigate its applicability in different imaging scenarios, the described assessment process was performed for both CT and MRI.

Three different (*3_15*, *3_20*, *5_20*) CT and one (*6_20*) MRI marker configuration were investigated. The number preceeding the underscore denotes the diameter of the fiducial features ($3{\mid }5{\mid }6$ mm), while the number following the underscore indicates the minimal distance between centroids of neighboring fiducial features ($15{\mid }20$ mm). As fiducial feature material we used steel balls (CT configurations) or the MRI capable *PinPoint*^©^*-187* spots (Beekley Corporation, Bristol, CT, USA).

Image acquisition was conducted with the Siemens *SOMATOM Definition Flash* CT scanner and with the *MAGNETOM Aera 1.5 Tesla* MRI scanner (Siemens Healthcare GmbH, Erlangen, BV, Germany). The details regarding the slightly different measurement setups for the CT/MRI assessment to account for the imaging specifics as well as the MRI image acquisition settings are provided in *Online Resource 2 and 6*. The assessment procedure described above was followed for each marker configuration and both phantoms. This resulted in a total number of 36 CT and 24 MRI scans (12$\times$ T1- and 12$\times$ T2-weighted). For both setups, additional six CT and six T1-weighted MRI images were acquired directly one after the other with the fiducial marker of the 3_15 (CT) or 6_20 (MRI) configuration permanently attached to a docking station to investigate the repeatability error of the experimental setup. These images were processed the same as described for all other CT/MRI volume images.

Each CT scan was reconstructed in the slice thicknesses and slice spacings $0.6{\mid }1{\mid }3{\mid }5$ mm (DICOM tags 0018, 0050 and 0018, 0088), respectively, with an in-plane voxel resolution of $0.98\times 0.98$ mm. In case of the MRI images, the voxel size was $0.5\times 0.5\times 1.0$ mm (T1-weighted) and $0.4\times 0.4\times 4.0$ mm (T2-weighted). For all marker configurations and slice thicknesses, the PPEs were then determined as described above for the virtual target point(s) of the *plane* and *angle* phantom. The repeatability error was calculated according to the PPE metric based on the corresponding six successive CT/MRI volume images. All image volume files of the CT and MRI measurements and the PPE evaluation software are published on the open science framework under the link https://osf.io/phyzt/.

### Abdominal phantom study: use case CT-to-US registration

**Fig. 6:**
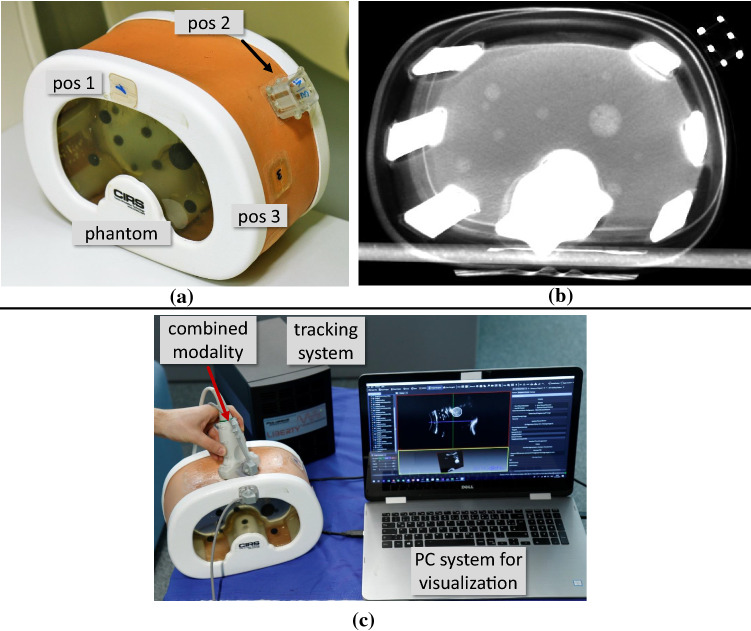
**a** Clipping positions used in the abdominal phantom study and for determining the clipping precision (section “Clipping precision”). **b** CT scan of the phantom with attached marker in a volume rendered view (rendering setting: VTK additive blend mode). **c** Setup for the CT-to-US registration

To estimate the target registration error (TRE) [18] when using the reattachable fiducial skin marker concept in clinical registration scenarios, we applied it in a realistic abdominal phantom study and conducted a CT-to-US registration.

For this purpose, we used a combined modality of a US probe and an EM tracking device, similar to the one proposed by *März et al.* [17], as shown in Fig. 6c. It was built by attaching the TX1 field generator (FG) (Polhemus Inc., Colchester, VT, USA) rigidly to the curved Telemed US probe (C4.5${\mid }$50${\mid }$128Z, Telemed Ltd., Vilnius, Lithuania). Determining the *US-to-tracking* transformation was performed as described by *März et al.* [17]. Further information about operating the combined modality and accessing the US images is provided in *Online Resource 3*.

The proposed workflow of Fig. 1 was then followed with the fiducial marker (3_15 configuration) consecutively attached to the three clipping positions of the abdominal phantom, previously used for determining the clipping precision (Fig. 6a and section “Clipping precision”), and a CT image (voxel size: $0.98 \times 0.98\times 1.0$ mm) was acquired for each position by the Siemens *SOMATOM Definition Flash* CT scanner. The *marker-to-CT* transformation was determined for all three CT images by applying the marker localization algorithm described in section “Fiducial marker localization in CT/MRI images”. Then, the upper ten ball-shaped targets of the phantom were manually segmented by the authors in all three CT images.

To register the CT segmentation data to the US space, the sensor holder with attached RX2 tracking sensor was clipped on one of the three clipping positions and the pose of the sensor holder relative to the combined modality was determined during US imaging (Fig. 6c). Based on the live tracking data and the known pose of the sensor holder relative to the fiducial marker as well as the previously determined *US-to-tracking* and *marker-to-CT* transformations, we calculated the overall transformation $T_{\mathrm{CT} \rightarrow \mathrm{US}}$ and superimposed the segmentation data on the 2D US image (see *Online Resource 3* for detailed information). The TRE was then determined for each target by calculating the Euclidean distance between the registered and the manually marked centroid of the target. Marking was performed by the authors with disabled registration overlay in the plain US image. Finally, we calculated the mean ($n = 10$) TRE and repeated the same procedure for all three clipping positions.

## Results

The *clipping precision* provided by the clipping plate was found to be 0.02 ± 0.01${\mid }$0.02 ± 0.02${\mid }$0.01 ± 0.01 mm ($\epsilon _{\mathrm{trans}}$) and 1.80$^{\circ }$ ± 0.25$^{\circ }{\mid }$ 0.87$^{\circ }$ ± 0.19$^{\circ }{\mid }$0.62$^{\circ }$ ± 0.22$^{\circ }$ ($\epsilon _{\mathrm{rot}}$) for the clipping positions 1${\mid }$2${\mid }$3, respectively. Furthermore, our proposed fiducial marker localization algorithm localized the fiducial marker in all ($n=201$) evaluated CT and MRI images.

**Table 1:**
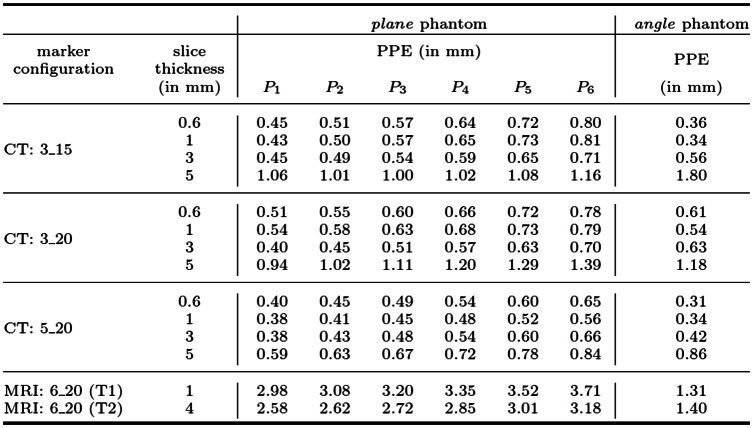
Evaluation results *plane* and *angle* phantom

The definition of the six virtual target points $P_{1,\ldots ,6}$ is given in Fig. 5

***Localization error*** In case of the CT experiments, the repeatability error was 0.08${\mid }$0.05${\mid }$0.20${\mid }$0.22 mm for the slice thicknesses 0.6${\mid }$1${\mid }$3${\mid }$5 mm, respectively. For the MRI setup, it was 0.06 mm (slice thickness: 1 mm). The PPEs of both the *plane* and *angle* phantom and all evaluated marker configurations are given in Table 1. Averaged over the four CT slice thicknesses, the mean ($n=4$) PPEs of the marker configurations 3_15${\mid }$3_20${\mid }$5_20 were determined to be 0.60${\mid }$0.60${\mid }$0.44 mm, respectively, for the *plane* phantom and 0.77${\mid }$0.74${\mid }$0.48 mm for the *angle* phantom (target distance: 100 mm).

In case of the MRI experiments, the mean ($n=2$) PPE was determined to be 2.78 mm for the *plane* and 1.36 mm for the *angle* phantom (target distance: 100 mm). Illustrative diagrams of these results are given in *Online Resource 2*.

***Abdominal phantom study*** In general, the visual superimposition of the performed CT-to-US registration matched the targets in the US image very well. Example snapshots are provided in *Online Resource 3*. This is in line with the evaluated mean ($n=10$) TREs, which were found to be 1.51 ± 0.75${\mid }$2.76 ± 0.91${\mid }$4.65 ± 1.22 mm for the clipping positions 1${\mid }$2${\mid }$3, respectively.

## Discussion

Fiducial markers are the default choice for a wide range of applications in clinical practice, such as neurosurgical interventions or punctures of abdominal lesions. However, as most of the fiducial markers, especially non-invasive skin markers, cannot be positioned reproducibly in the same pose, pre-interventional imaging has to be performed immediately before the intervention for fiducial marker-based registrations.

To overcome these limitations, we proposed a non-invasive, reattachable fiducial marker that allows reproducible positioning by means of a plaster clip-on mechanism and that can be automatically localized in different imaging modalities. We systematically examined individual parts of the marker concept and found that the clipping precision provided by the clipping mechanism was essentially not affected by the mounting of the plaster and that the marker could successfully be localized for conventional imaging settings. In addition, we conducted an abdominal phantom study to investigate the overall TRE when the whole marker concept is applied in a multi-modal interventional registration scenario.

The TREs determined in this study appear to be inversely correlated with the angular *clipping precision* $\epsilon _{\mathrm{rot}}$ of the individual clipping positions 1${\mid }$2${\mid }$3, even though it might be expected that the worse the angular clipping precision, the larger the TRE. We assume that the increase of the TRE for the clipping positions 2 and 3 was mainly caused by the different tracking technologies used in both experiments: For determining the clipping precision we used an optical tracking system, whereas an EM tracking system was utilized in the abdominal phantom study. In case of EM tracking systems, however, the tracking error may increase with an increased distance between the FG and the EM sensor. The mean distance between the combined modality and the EM sensor was largest (approx. 25 cm) in case of clipping position 3 and smallest (approx. 10 cm) for clipping position 1. Additionally, the rigid cable of the EM sensor used in the phantom study led to a well observable tilting of the sensor holder during the registration in case of the skin-mimicking mounting conditions of the clipping positions 2 and 3, whereas tilting was prevented due to the rigid mounting conditions in case of clipping position 1. This and the increased tracking error could be the reasons for the worse TRE values of the clipping positions 2 and 3. We thus recommend to place the *clipping plate*, and with this the fiducial marker and the sensor holder, as close as possible to the target and to additionally tape the cable of the EM sensor to the skin.

Even though, out of practical reasons, our localization assessment sampled the quite high-dimensional space of possible marker poses within the intervention volume only sparsely and did not directly measure the fiducial localization error, the achieved low prediction errors below 0.6 mm (CT) and 1.4 mm (MRI) in the optimal case indicate that accurate localization of the fiducial marker in different imaging modalities can be achieved. For localizing the marker it was required that its fiducial features were completely captured in the CT/MRI volume image. This was ensured by configuring the slice thickness and slice spacing (DICOM tags 0018,0050 and 0018,0088) such that they had the same value. With this setting, fiducial marker localization was successful in all imaging settings of our assessments, which is comparable to findings in other studies [5, 22, 26]. However, robustness remains to be shown for in-vivo settings. An MRI assessment on probands could be a first step.

The accuracy and precision required by a fiducial marker-based system for being applicable in clinical workflows mainly depends on the specific use case. Navigating, e.g., to *measureable* abdominal tumor lesions $\ge$ 10 mm according to the RECIST guideline [4] by means of the new marker concept might be possible with sufficient accuracy. Given the low TREs (1.51 mm  ±  0.75 mm, optimal marker positioning) reported in our abdominal phantom study, serious considerations should be made to plan further steps to use the new marker concept in real clinical interventions in the future. Particularly compelling is the automatic and fast nature of the registration approach that is of great importance in intraoperative applications. Non-rigid settings that are subject to, e.g., organ motion due to respiration are currently not covered by the proposed approach and require future investigation of motion compensation methods or application of respiratory gating techniques.

## Conclusion

The reattachable fiducial skin marker concept allows reproducible positioning of the marker on a realistic abdominal phantom and automatic localization in different imaging modalities. The achieved low TREs indicate the potential applicability of the marker concept for clinical interventions, such as the puncture of abdominal lesions, where current image-based registration approaches still lack robustness and existing marker-based methods are often impractical.

## Supplementary information

The supplementary files provided online are as follows:

- Online Resource 1: Marker design and localization algorithm details
- Online Resource 2: Marker assessment details
- Online Resource 3: Abdominal phantom study details
- Online Resource 4: 3D design file *plane* phantom
- Online Resource 5: 3D design file *angle* phantom
- Online Resource 6: Acquisition settings MRI measurements

## Supplementary Information

Below is the link to the electronic supplementary material.

Supplementary file 1 (pdf 1846 KB)

Supplementary file 2 (pdf 3090 KB)

Supplementary file 3 (pdf 853 KB)

Supplementary file 4 (stl 609 KB)

Supplementary file 5 (stl 335 KB)

Supplementary file 6 (pdf 136 KB)

## Data Availability

All image volume files of the CT and MRI measurements are published on the open science framework under the link https://osf.io/phyzt/.

## Code availability

See MITK tag ReattachableFiducialSkinMarkerIJCARS2022.04 (https://github.com/BMittmannTest/MITK/tree/ReattachableFiducialSkinMarkerIJCARS2022.04).
