# Supplementary material for: Reattachable fiducial skin marker for automatic multimodality registration
Source: Int J Comput Assist Radiol Surg. 2022 May 23;17(11):2141–50. doi: 10.1007/s11548-022-02639-7 (PMC9515062; doi:10.1007/s11548-022-02639-7)
Supplement: Supplementary file 1 — (pdf 1846 KB) [file 11548_2022_2639_MOESM1_ESM.pdf]

# Marker design and localization algorithm details

As described in the paper and illustrated in Figure 1, the proposed fiducial skin marker concept consists of three components: the *clipping plate*, the *fiducial marker* and the *sensor holder*. These three components are applicable in multi-modal registration workflows of image guided interventions as described in the next section. Furthermore, we would like to give specific insights regarding the design of the fiducial marker itself (component 2), because this is relevant for the fiducial marker localization algorithm. Both the marker specific design insights as well as the localization algorithm details are described in the following sections.

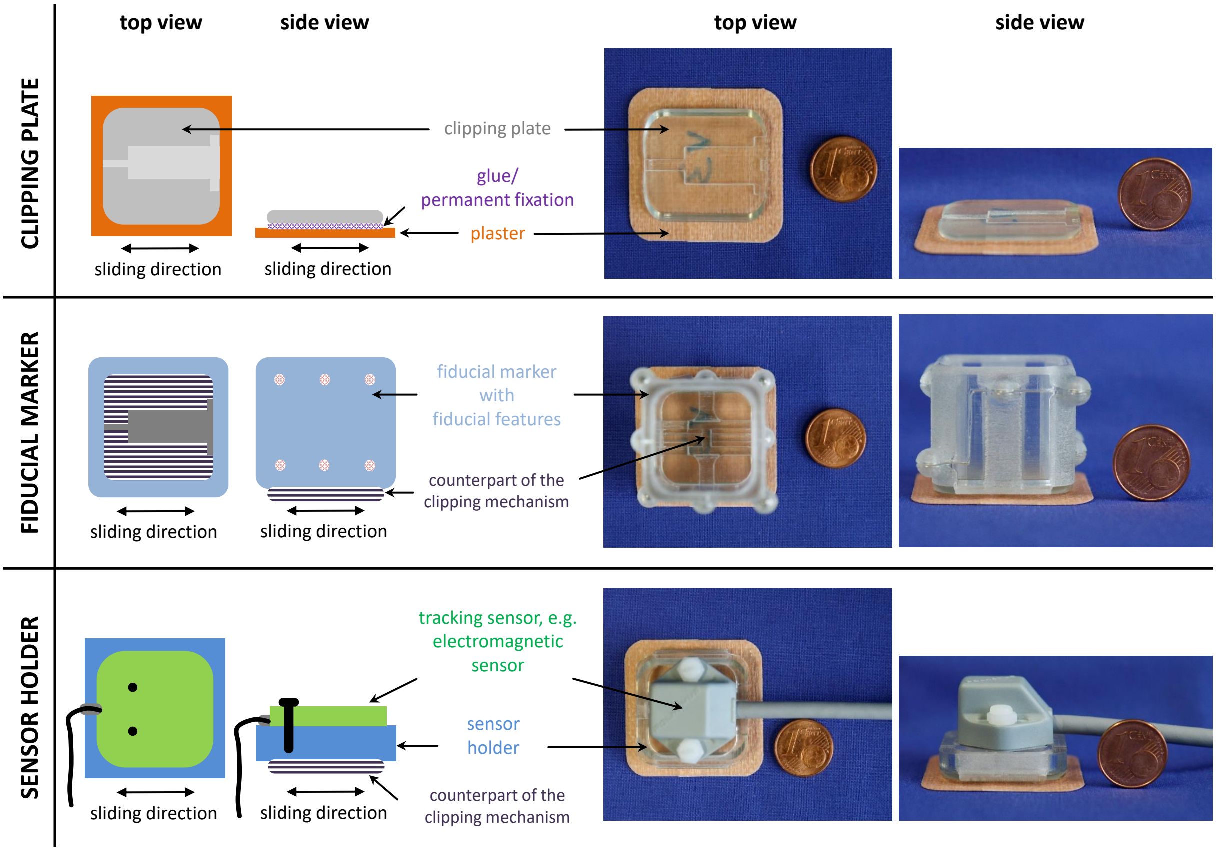


Figure 1: Proposed reattachable fiducial skin marker concept consisting of a clipping plate (component 1), a fiducial marker (component 2) and a sensor holder (component 3). The fiducial marker and the sensor holder are reproducibly (re)attachable on the clipping plate.

Mittmann, B. J., Seitel, A., Echner, G., Johnen, W., Gnirs, R., Maier-Hein, L., Franz, A. M.: **Reattachable fiducial skin marker for automatic multimodality registration**, *Int J Comput Assist Radiol Surg*, Corresponding author: A. M. Franz (alfred.franz@thu.de), Department of Computer Science, Ulm University of Applied Sciences, Albert-Einstein-Allee 55, Ulm, 89081, BW, Germany.

## Usage for multimodality registration in image guidance systems

As preparatory step, the clipping plate is attached with a standard plaster on the patient and the fiducial marker is mounted on it (Figure 2). After pre-interventional computed tomography (CT) or magnetic resonance (MR) imaging, the fiducial marker is localized in the image and the transformation from the fiducial marker coordinate system (CS) to the image CS is calculated by using, for instance, the point-based method proposed by *Horn* (Horn 1987). The fiducial marker can then be detached, but the clipping plate remains on the patient till the registration workflow is completed. Immediately before the intervention, the sensor holder is mounted on the clipping plate. The sensor holder provides a base for mounting an electromagnetic (EM) sensor or an optical marker that allows localizing interventional instruments, such as a trackable needle or an ultrasound (US) probe, spatially in relation to the sensor holder. The information obtained from all three components as well as from medical imaging can then be used to generate supportive augmented reality information for the physician during the intervention, such as superimposing a needle relative to a US image and additionally fading in segmentation structures from CT or MR imaging.

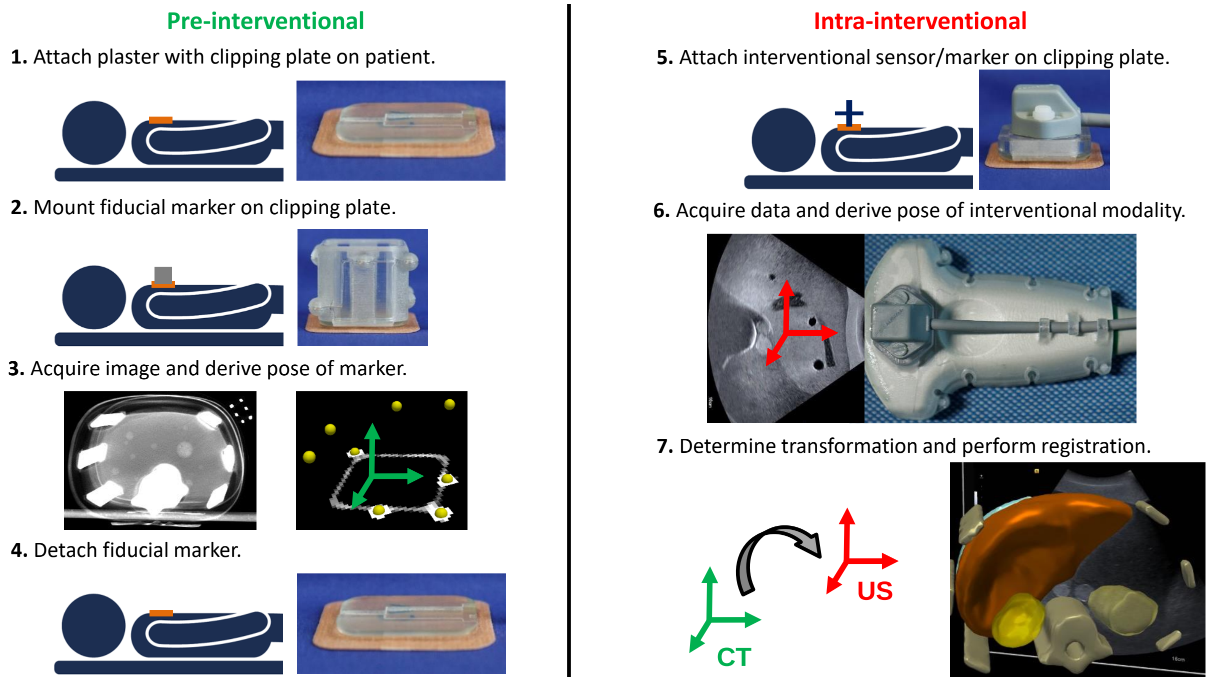


Figure 2: Proposed workflow of a non-invasive, reattachable fiducial skin marker applicable for multi-modal interventional registration approaches.

## Fiducial marker: design specific insights

The fiducial marker was developed based on the recommendations given by *Fitzpatrick et al.* (Fitzpatrick et al. 1998). Accordingly, we designed the fiducial marker to contain eight spherical, exchangeable fiducial features, which were required to be distributed over two parallel planes with four fiducial features in each plane. This configuration allows identifying and automatically enumerating each fiducial feature based on the known Euclidean distances to the other seven fiducial features (Figure 3) and decreasing the influence of leverage effects on the registration error compared to single-plane fiducial feature distributions (Kingma et al. 2011). The minimal distance $d$ between centroids of neighboring fiducial features was either 15 mm or 20 mm in our experiments described in the paper. A minimal distance below 15 mm prevented the automatic localization of the marker in CT images with a slice thickness of 5 mm, as some of the fiducial features were depicted as a joined, non-separable image structure caused by the partial volume effect. Due to the exchangeability of the fiducial features, the fiducial marker can be adapted to different imaging modalities, such as CT or MRI.

Furthermore, we would like to mention that the fiducial marker as well as the clipping plate and the sensor holder were all printed with the 3D printer *Objet500 Connex3* (Stratasys Ltd., Eden Prairie, MN, USA) by using the company's own material *VeroClear*.

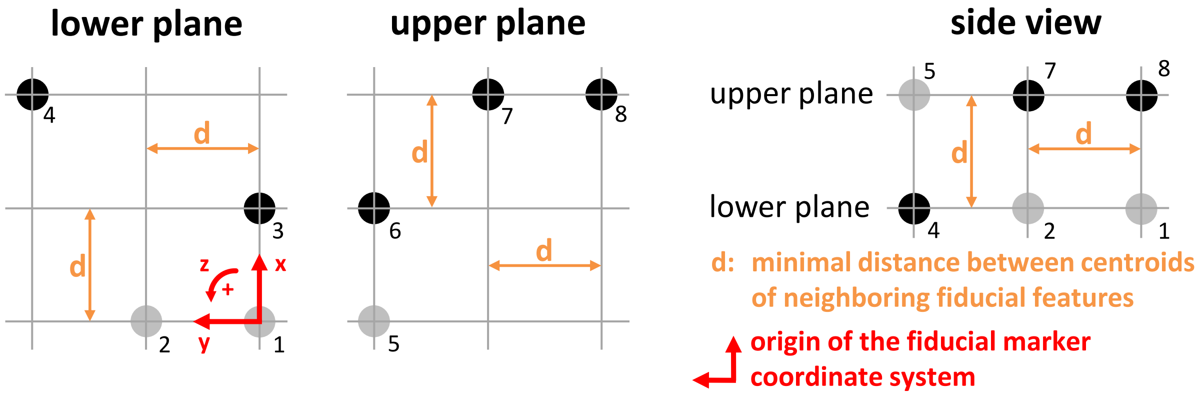


Figure 3: Fiducial feature distribution: the eight spherical fiducial features are indicated as black and gray circles. They are distributed over two parallel planes. The distance between the planes is equal to the minimal distance $d$ between centroids of neighboring fiducial features. Identifying and enumerating the fiducial features during the marker localization process is based on the Euclidean distance configuration of the fiducial features' centroids. Fiducial feature number 1, e.g., has the following distance configuration sorted in ascending order: d, d, $\sqrt{5}$·d, $\sqrt{5}$·d, $\sqrt{6}$·d, $\sqrt{6}$·d, $\sqrt{8}$·d.

## Fiducial marker localization – algorithm details

As described in the paper, detecting fiducial feature candidates within an imaging volume is based on an ~~ITK~~ Insight Toolkit (ITK) filter pipeline (Figure 4) that is configured to be self-adjustable depending on the image input (CT or MRI volume image). The algorithm was implemented in the Medical Imaging Interaction Toolkit (Nolden et al. 2013) including the ITK and the VTK libraries.

In detail, the image is processed by the ITK *ThresholdImageFilter*, first. It discards soft tissue signals and extracts components in the image that are in the brightness range of the fiducial features (Figure 4i-ii). The upper threshold is 3,200 for CT and 1,800 for MRI images. The lower threshold (CT: 500, MRI: $0.05 \cdot$ maximum voxel value of image) is chosen with respect to the partial volume effect, which decreases the voxel values of the fiducial features' edge areas, notably in images with a coarser ($\geq 3$ mm) slice thickness. As the fiducial features' edges are image regions with a large gradient of the voxel values, these can be detected by a second derivative image filter. For this task, we apply the ITK *LaplacianRecursiveGaussianImageFilter* as edge detection filter (Figure 4iii). To obtain the fiducial features as bright blob regions again we apply this filter twice (Figure 4iv). This simplifies the further localization steps, as the centroid of these blob regions can

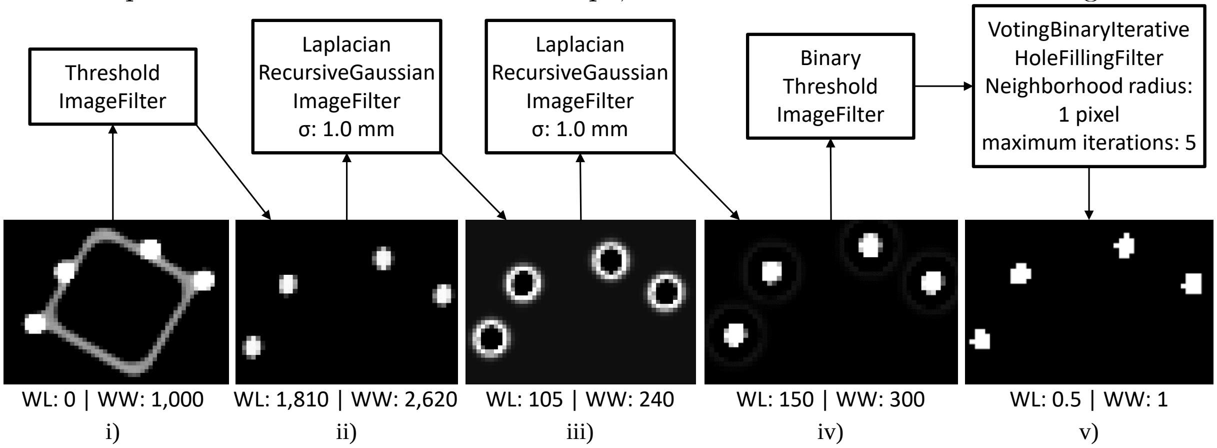


Figure 4: ITK filter pipeline for identifying fiducial feature candidates. The pictures i) - v) illustrate the output of the image filtering steps when using a CT image of the fiducial marker. i) The fiducial features are depicted as bright blob regions. ii) Output of the *ThresholdImageFilter*. iii) Output of the *LaplacianRecursiveGaussianImageFilter* applied once. iv) Output of the same filter applied twice. v) Contiguous white voxel regions are treated as fiducial feature candidates. As some of the true candidates might rarely contain black voxels with a value of zero, these are filled up by the ITK *VotingBinaryIterativeHoleFillingFilter*, finally. WL = window level, WW = window width.

Mittmann, B. J., Seitel, A., Echner, G., Johnen, W., Gnirs, R., Maier-Hein, L., Franz, A. M.: **Reattachable fiducial skin marker for automatic multimodality registration**, *Int J Comput Assist Radiol Surg*, Corresponding author: A. M. Franz (alfred.franz@thu.de), Department of Computer Science, Ulm University of Applied Sciences, Albert-Einstein-Allee 55, Ulm, 89081, BW, Germany.

be easily retrieved by means of the ITK library. Afterwards, non-fiducial-feature image regions having a low gradient of the voxel values are suppressed by applying the ITK *BinaryThresholdImageFilter* that binarizes the image. Its thresholds depend on the voxel value characteristics of the imaging modalities. A fixed threshold range between 350 and 10,000 HU is chosen for CT images. However, contrary to CT imaging, the range of voxel values in MRI images varies depending on the MRI acquisition settings and the quantity of hydrogen protons contained in the field of view, making fixed thresholds inappropriate. Hence, the lower threshold of this filter is dynamically adjusted and is 2, 10, 15 and 20 for a maximum image voxel value $\in [-\infty;150[$, $[150;300[$, $[300;399]$ and $]399;+\infty]$, respectively, while keeping the upper threshold constant at 500 for MRI images. All voxels within the threshold range are set to one (white voxels), while all other voxels are set to zero (black voxels). We treat all contiguous white voxel regions as fiducial feature candidates. Eliminating false candidates and determining the pose of the fiducial marker in the 3D imaging volume is then done following a three-step procedure:

1. Fiducial feature candidates are identified in a label map using the ITK *BinaryImageToShapeLabelMapFilter*. The centroid of each candidate is calculated.

2. The candidate list is pruned by excluding candidates with a volume smaller than 0.6 times the real fiducial feature volume as well as iteratively excluding candidates for which no known distance configuration (Figure 3) applies. Additionally, candidates greater than two times the real fiducial feature volume are rejected in case of MRI images, too.

3. Based on the ordered, remaining fiducial feature candidates according to the pairing procedure described below, the pose of the fiducial marker is determined by calculating the transformation from the fiducial marker CS to the image CS by using the point-based method proposed by *Horn* (Horn 1987). If less than eight fiducial features are identified in step two, the fiducial marker may not be localized successfully depending on which fiducial features are missing.

Mittmann, B. J., Seitel, A., Echner, G., Johnen, W., Gnirs, R., Maier-Hein, L., Franz, A. M.: **Reattachable fiducial skin marker for automatic multimodality registration**, *Int J Comput Assist Radiol Surg*, Corresponding author: A. M. Franz (alfred.franz@thu.de), Department of Computer Science, Ulm University of Applied Sciences, Albert-Einstein-Allee 55, Ulm, 89081, BW, Germany.

### *Pairing procedure of the localized fiducial features*

Ordering and paring the localized fiducial features in the CT/MRI volume image with their model counterparts, as defined in Figure 3, is based on the known fiducial feature distance configuration. The order, in which the fiducial features are identified, is as follows:

- **Fiducial number 1 and 4:** According to Figure 3, both fiducial number 1 and 4 have a unique distance configuration to the other seven fiducial features. For fiducial number 1 it is: d, d, $\sqrt{5}$·d, $\sqrt{5}$·d, $\sqrt{6}$·d, $\sqrt{6}$·d, $\sqrt{8}$·d. For fiducial number 4 it is: $\sqrt{2}$·d, $\sqrt{2}$·d, $\sqrt{5}$·d, $\sqrt{5}$·d, $\sqrt{5}$·d, $\sqrt{5}$·d, $\sqrt{8}$·d. Hence, these unique distance configurations are used to identify fiducial number 1 and 4, first.
- **Fiducial number 2 and 3:** Fiducial number 2 and 3 have the lowest Euclidean distance to fiducial number 1. They can be determined by comparing the Euclidean distances between fiducial number 1 and the remaining six fiducial features. Having determined the two fiducial features with the lowest distance to fiducial number 1, the cross product of the x and y vector (Figure 3) from fiducial number 1 to both fiducial features is calculated. Based on the pointing direction of the cross product, it can then be decided which of the two fiducial features corresponds to fiducial number 2 and to fiducial number 3.
- **Fiducial number 5:** Fiducial number 5 is then the fiducial feature with the closest distance to fiducial number 2.
- **Fiducial number 8:** Fiducial number 8 is the fiducial feature with the closest distance to fiducial number 3.
- **Fiducial number 6:** Fiducial number 6 is the fiducial feature with the closest distance to fiducial number 5.
- **Fiducial number 7:** Fiducial number 7 is the fiducial feature with the closest distance to fiducial number 8.

Mittmann, B. J., Seitel, A., Echner, G., Johnen, W., Gnirs, R., Maier-Hein, L., Franz, A. M.: **Reattachable fiducial skin marker for automatic multimodality registration**, *Int J Comput Assist Radiol Surg*, Corresponding author: A. M. Franz (alfred.franz@thu.de), Department of Computer Science, Ulm University of Applied Sciences, Albert-Einstein-Allee 55, Ulm, 89081, BW, Germany.

## References

Fitzpatrick JM, West JB, and Maurer CR (1998) Predicting error in rigid-body point-based registration. IEEE Transactions on Medical Imaging 17(5): 694–702.

Horn BKP (1987) Closed-form solution of absolute orientation using unit quaternions. Journal of the Optical Society of America A, 4(4): 629–642. https://doi.org/10.1364/JOSAA.4.000629

Kingma R, Rohling RN, Nguan C (2011) Registration of CT to 3D ultrasound using near-field fiducial localization: A feasibility study. Computer Aided Surgery 16(2): 54–70. https://doi.org/10.3109/10929088.2011.556181

Nolden M, Zelzer S, Seitel A, Wald D, Müller M, Franz AM, Maleike D, Fangerau M, Baumhauer M, Maier-Hein L, Maier-Hein KH, Meinzer HP, and Wolf I (2013) The Medical Imaging Interaction Toolkit: challenges and advances. International Journal of Computer Assisted Radiology and Surgery 8(4): 607–620.

 Corresponding author: A. M. Franz (alfred.franz@thu.de), Department of Computer Science, Ulm University of Applied Sciences, Albert-Einstein-Allee 55, Ulm, 89081, BW, Germany.
