# Supplementary material for: Reattachable fiducial skin marker for automatic multimodality registration
Source: Int J Comput Assist Radiol Surg. 2022 May 23;17(11):2141–50. doi: 10.1007/s11548-022-02639-7 (PMC9515062; doi:10.1007/s11548-022-02639-7)
Supplement: Supplementary file 2 — (pdf 3090 KB) [file 11548_2022_2639_MOESM2_ESM.pdf]

Marker assessment details

As described in the paper, we investigated two main aspects for assessing the fiducial marker: the *clipping precision* provided by the clipping mechanism and the *localization error* of the marker itself (component 2). In the following sections, we will describe the details and some specific insights regarding:

1. The technical implementation of the clipping precision experiment
2. The high precision assessment phantoms
3. The *point prediction error* (PPE) used for evaluating the localization error
4. The experimental setup to determine the localization error in CT images
5. The experimental setup to determine the localization error in MRI images

Furthermore, we give:

6. Illustrative diagrams of the PPE evaluation results
7. Additional remarks regarding the PPE results

Mittmann, B. J., Seitel, A., Echner, G., Johnen, W., Gnirs, R., Maier-Hein, L., Franz, A. M.: **Reattachable fiducial skin marker for automatic multimodality registration**, *Int J Comput Assist Radiol Surg*, Corresponding author: A. M. Franz (alfred.franz@thu.de), Department of Computer Science, Ulm University of Applied Sciences, Albert-Einstein-Allee 55, Ulm, 89081, BW, Germany.

1 Clipping precision

As mentioned in the main article, we investigated rigid and skin-mimicking mounting conditions provided by the *Image-Guided Abdominal Biopsy Phantom, Model 071B* (Computerized Imaging Reference System, Inc., Norfolk, VA, USA), as illustrated in Figure 1. In case of clipping position 1, only the clipping plate was stuck on the rigid plastic frame of the phantom. Skin-mimicking mounting conditions were simulated by attaching the clipping plate with a plaster (*DracoPor 3.8 x 3.8 cm*, Dr. Ausbüttel & Co. GmbH, Witten, NRW, Germany) to the soft, skin-like phantom surface in case of the clipping positions 2 and 3.

For each position, we clipped the sensor holder with a rigidly attached passive, optically localizable sensor 40 times on and off the clipping plate and determined the ~~position and the~~ pose of the sensor holder in its mounted state with respect to a simultaneously localized reference sensor (Figure 1). Spatially localizing both passive sensors was performed with the Micron Tracker 2 (ClaroNav, Toronto, ON, Canada). It was chosen due to its lower tracking error compared to electromagnetic tracking systems (Franz et al. 2014). The reference sensor was required to be able to compensate well observable temporal measurement data drifts of the Micron Tracker 2 tracking system. For each of the 40 measurements of one clipping position, we recorded the sensor ~~positions and~~ poses with 100 measurement samples and averaged (n = 100) the measurement data of each sensor for calculating ϵ_{trans} and ϵ_{rot} as described in the paper. This resulted in mean $\epsilon_{trans} \mid \epsilon_{rot}$ values below 0.02 mm|1.80 degrees, respectively.

Compared with the tracking error (0.20 mm) of the Micron Tracker 2 in the center of its field of measurement (Micron2006), the determined uncertainties of the clipping precision are low. Based on our method of removing the mean from the measurements, it is assumed that the tracking error associated with the tracker is mitigated but not eliminated completely. Nevertheless, the reported low ϵ_{trans} and ϵ_{rot} values indicate that accurate (re-)attachement of the fiducial marker and the sensor holder on the clipping plate is possible.

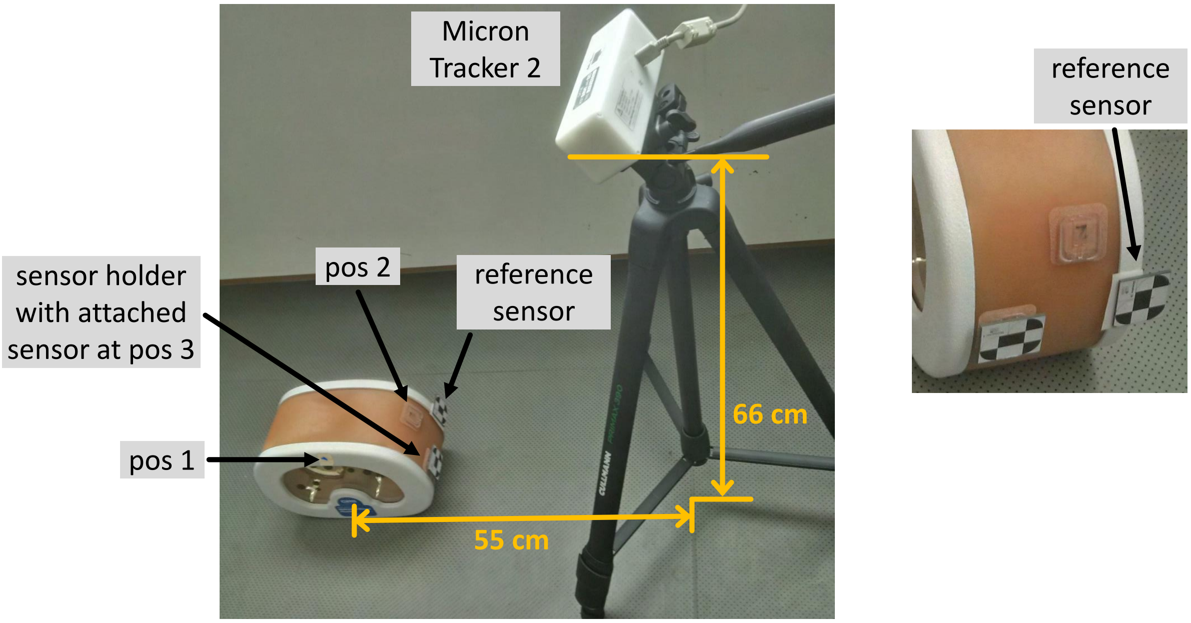

a) b)

Figure 1: a) Experimental setup of the clipping precision experiment. As illustrated, the middle of the phantom had a distance of 55 cm to the vertical central rod of the tripod and the distance of the Micron Tracker 2 to the floor was approximately 66 cm. In this setting, the phantom and the attached optically trackable sensors were placed at approximately 85 cm from the tracking camera of the Micron Tracker 2, and thus in the middle of its specified field of measurement. Please note: The field of measurement of the Micron Tracker 2 is pyramid-shaped (height of pyramid: 120 cm) with the top of the pyramid starting 15 cm in front of the tracker (Micron2006). At the bottom, it is spherically closed. The clipping positions (pos 1|2|3) of this experiment were the same as in the abdominal phantom study. b) Detailed view showing the physical relationship between the sensor holder with an attached optically localizable sensor and the reference sensor. In this image, the sensor holder was placed at clipping position 3.

Mittmann, B. J., Seitel, A., Echner, G., Johnen, W., Gnirs, R., Maier-Hein, L., Franz, A. M.: **Reattachable fiducial skin marker for automatic multimodality registration**, *Int J Comput Assist Radiol Surg*, Corresponding author: A. M. Franz (alfred.franz@thu.de), Department of Computer Science, Ulm University of Applied Sciences, Albert-Einstein-Allee 55, Ulm, 89081, BW, Germany.

2 Localization error – high precision assessment phantoms

As explained in the paper, the two high precision measurement phantoms (Figure 2) serve as a mechanical platform to investigate marker placement at predefined poses in an imaging volume roughly reflecting realistic intervention scenarios. Both phantoms were printed by the 3D printer *Objet500 Connex3* (Stratasys Ltd., Eden Prairie, MN, USA). For mounting the fiducial marker easily on the docking stations, each station was equipped with the clipping plate (component 1) of the reattachable fiducial skin marker concept. Furthermore, we did not remove the proton-rich support material of the docking stations of the *angle* phantom and we designed the *plane* phantom to self-provide a waterfillable bowl in order to increase the amount of hydrogen protons in the field of view (FOV) in case of MRI measurements. Additionally, the water filled bowl could be used as water bath for registration experiments involving ultrasound. The 3D design files of the phantoms are provided as *Online Resource 4a* and *4b*.

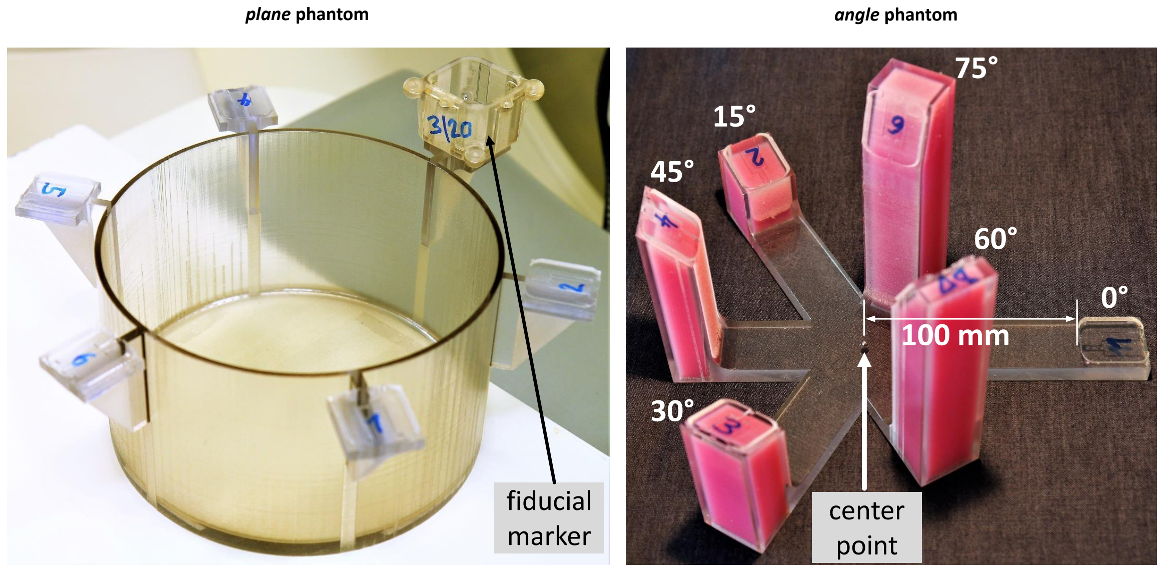

Figure 2: Pictures of the *plane* and *angle* phantom. The relative Euclidean distance between the symmetry point and any docking station of the phantoms is 100 mm.

Mittmann, B. J., Seitel, A., Echner, G., Johnen, W., Gnirs, R., Maier-Hein, L., Franz, A. M.: **Reattachable fiducial skin marker for automatic multimodality registration**, *Int J Comput Assist Radiol Surg*, Corresponding author: A. M. Franz (alfred.franz@thu.de), Department of Computer Science, Ulm University of Applied Sciences, Albert-Einstein-Allee 55, Ulm, 89081, BW, Germany.

3 Localization error – *point prediction error* (PPE)

As explained in the paper, the *point prediction error* (PPE) is a measure of fiducial marker localization that is not dependent on reference annotation variability due to inter-observer variability and image resolution. To determine the PPE, we followed a four step procedure:

1. The fiducial marker was mounted consecutively to each docking station and one volume image was acquired of each marker pose.
2. The fiducial marker localization algorithm described in the paper was applied on each image resulting in the transformations $T_1, ..., T_6$ from the marker coordinate system (CS) to the image CS.
3. The transformations $T_1, ..., T_6$ were applied to the virtual target point(s), as defined in Figure 3, resulting in a set of six transformed points $p_1, ..., p_6$ for each virtual target point.
4. The PPE was calculated according to Equation 1 for each virtual target point. To prevent any impact of inter-rater reliability on the evaluation results, we automated the PPE calculations in the MITK (see branch T26573-FiducialBasedMRIregistration).

$$PPE = \sqrt{\frac{\sum_{i=1}^{6}(p_{i,x} - \bar{p}_x)^2 + (p_{i,y} - \bar{p}_y)^2 + (p_{i,z} - \bar{p}_z)^2}{6}} \tag{1}$$

p_i	coordinate vector of the ith transformed point
$\bar{p}$	coordinate vector of the approximated symmetry point, i.e. mean $(p_1, ..., p_6)$
$\bar{p}_x$	scalar x-coordinate of $\bar{p}$. $\bar{p}_y$ and $\bar{p}_z$ accordingly

As the true position of the phantom's symmetry point could not be determined automatically and with high precision in the image CS independent of the slice thickness, we made use of the point symmetry of the phantoms and calculated the mean center point $\bar{p}$ of the transformed points $p_1, ..., p_6$ as approximation. To verify this assumption, we placed a 3 mm spherical steel ball at the symmetry point and manually measured the distance between the approximated symmetry point $\bar{p}$ and the center point of the steel

 Corresponding author: A. M. Franz (alfred.franz@thu.de), Department of Computer Science, Ulm University of Applied Sciences, Albert-Einstein-Allee 55, Ulm, 89081, BW, Germany.

ball marked in the CT image. This resulted in deviations below 0.1 mm, which justifies our approximation of the symmetry point.

The PPE was evaluated for both phantoms, resulting in the PPE_{plane} and the PPE_{angle}. The PPE_{plane} was used to specify the influence of the localization error with respect to different distances to virtual targets. Although, out of practical reasons, only six angulated marker poses were investigated in this high-dimensional space of possible angulated marker poses, the PPE_{angle} indicated, whether the angulated marker placement had an additional impact on the localization error. Furthermore, the PPE could be used to compare different fiducial markers or localization algorithms with respect to their localization error.

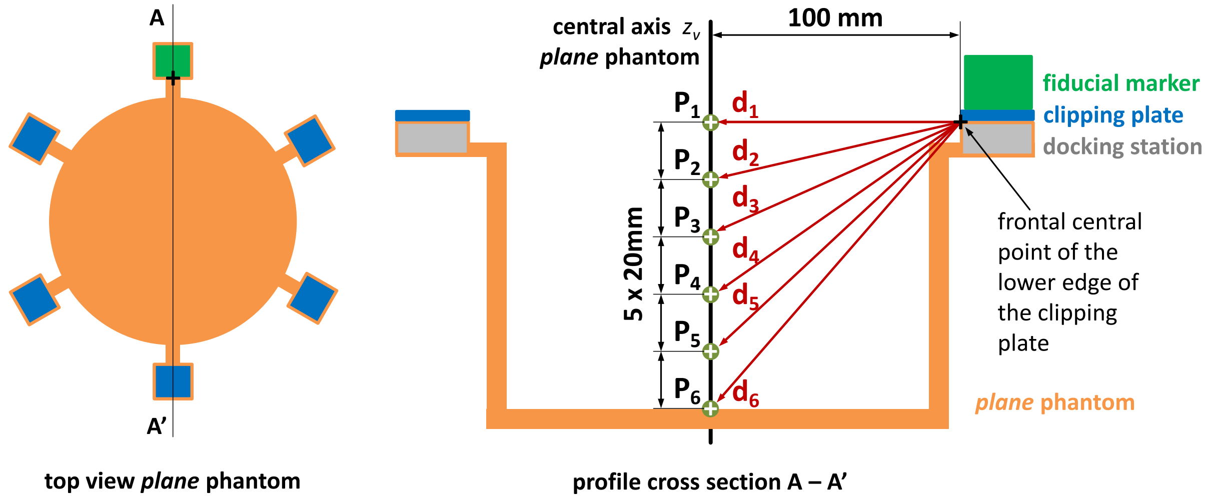

Figure 3: Illustration of the positions of the six virtual target points $P_{1,...,6}$, which were considered for assessing the fiducial marker in case of the *plane* phantom. The six Euclidean distances $d_1, ..., d_6$ to the target points are determined with respect to the frontal central point of the lower edge of the clipping plate: $d_1 = 100.00$ mm; $d_2 = 101.98$ mm; $d_3 = 107.70$ mm; $d_4 = 116.62$ mm; $d_5 = 128.06$ mm; $d_6 = 141.42$ mm. Due to its angular geometry, only one virtual target point (target distance: 100 mm) was considered in case of the *angle* phantom.

4 Localization error – experimental CT setup

As described in the paper, we investigated three different marker configurations (Table 1) with respect to their localization error in CT images. For this purpose, the *plane* and *angle* phantom were rigidly attached to a flat wooden panel placed on the CT bench, as illustrated in Figure 4a. The assessment procedure described in the paper was then followed for each marker configuration. Furthermore, we investigated the repeatability error of the experimental setup by acquiring additional six CT scans of the plane phantom directly one after the other with the fiducial marker of the 3_15 configuration permanently attached to a docking station without changing or hitting anything of the experimental setup. We further processed these six CT scans the same way, as described in the paper, and calculated the repeatability error according to the PPE metric, i.e. transforming the virtual target point from the fiducial marker CS to the image CS for each of the six images, determining the mean point and calculating the root mean squared distance between these points according to Equation 1 in section 3 of this document.

Characteristics of marker configurations

	**3_15**	**3_20**	**5_20**
diameter of metal fiducial features	3 mm	3 mm	5 mm
minimal distance between centroids of fiducial features	15 mm	20 mm	20 mm
total weight	10g	20g	24g

Table 1: Characteristics of the three marker configurations used in the study.

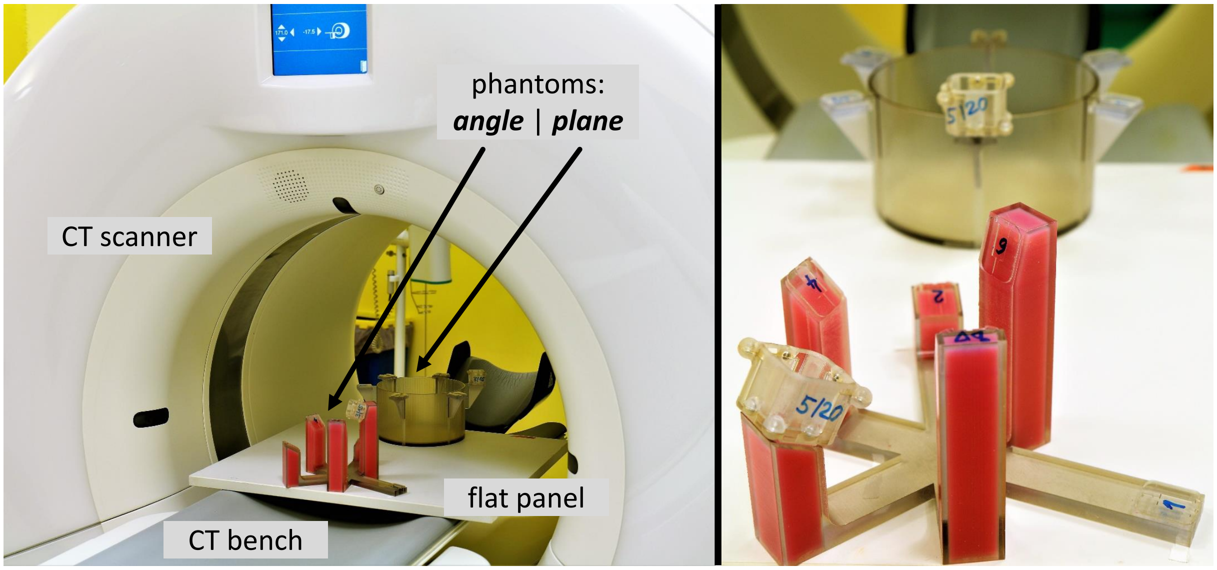

a) b)

Figure 4: Experimental setup of the CT measurements: a) The *plane* and the *angle* phantom were attached rigidly to a flat wooden panel, which was placed on the CT bench. b) Two identical fiducial markers were mounted separately to corresponding docking stations of each phantom in order to acquire a CT scan of the *plane* phantom and a CT scan of the *angle* phantom in one scanning procedure.

5 Localization error – experimental MRI setup

Contrary to CT imaging, the field of view (FOV) is preferably small in case of MRI. The larger the FOV is, the longer the scanning time and the greater the geometric image distortions are with increased distance to the isocenter, the origin of the magnetic field of the MRI scanner (Nejad-Davarani et al. 2019). Hence, to assess the localization error of the 6_20 marker configuration, we processed both phantoms separately and attached either the *plane* or the *angle* phantom rigidly to a flat wooden panel, which we placed on the MRI bench, as illustrated in Figure 5a. Image acquisition was conducted with the *MAGNETOM Aera 1.5 Tesla* MRI scanner (Siemens Healthcare GmbH, Erlangen, BV, Germany) in combination with the Siemens *Body 18* coil. To arrange the coil as close as possible around the phantom without hitting it, we had to place a wooden support frame next to the phantom (Figure 5). Furthermore, we positioned a small water filled box in the middle of the *angle* phantom and we filled the bowl of the *plane* phantom with water to make MRI acquisition possible by increasing the quantity of hydrogen protons in the FOV. To reduce image distortions caused by the chemical shift, we chose a bandwidth of 200 Hz/pixel and selected the other MRI acquisition settings in accordance with the acquisition settings of MRI radiation treatment planning sequences (see *Online Resource 5*).

By following the assessment procedure described in the paper, we acquired T1- and T2-weighted MRI images of each marker pose and both phantoms, resulting in a total of 24 MRI images (12x T1 and 12x T2). Additional six T1-weighted MRI images of the *angle* phantom were taken directly one after the other with the fiducial marker permanently attached to the first docking station without changing or hitting anything of the experimental setup to determine the repeatability error of the experimental setup. We evaluated these six MRI images the same way, as described above for the CT scans. The voxel size was 0.5 x 0.5 x 1.0 mm for all T1-weighted images and 0.4 x 0.4 x 4.0 mm for all T2-weighted images. The difference in the voxel size was related to image acquisition settings to minimize the scanning time that was approximately six minutes for one T1-weighted scan and ten minutes for one T2-weighted scan.

Mittmann, B. J., Seitel, A., Echner, G., Johnen, W., Gnirs, R., Maier-Hein, L., Franz, A. M.: **Reattachable fiducial skin marker for automatic multimodality registration**, *Int J Comput Assist Radiol Surg*, Corresponding author: A. M. Franz (alfred.franz@thu.de), Department of Computer Science, Ulm University of Applied Sciences, Albert-Einstein-Allee 55, Ulm, 89081, BW, Germany.

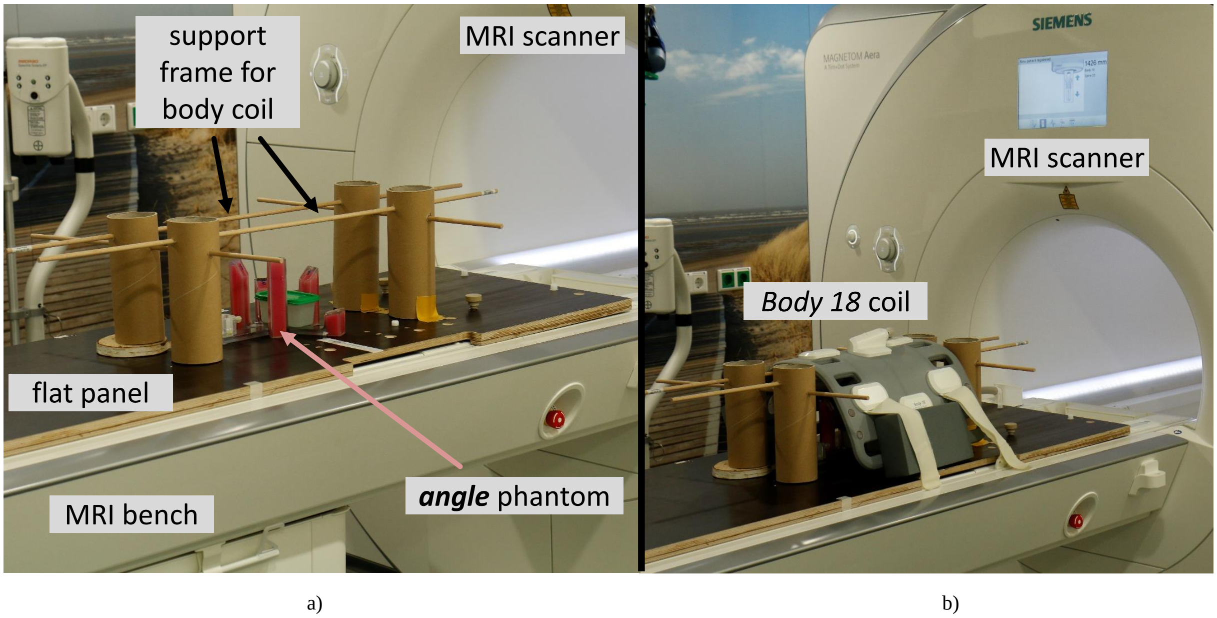

Figure 5: Experimental setup of the MRI measurements based on the assessment protocol. a) The image shows the *angle* phantom attached rigidly to a flat wooden panel, which was placed on the MRI bench. Additionally, a support frame for the body coil was placed on the panel. It allowed the body coil to be arranged as close as possible around the phantom without hitting it. b) Same setup but now with the *Body 18* coil placed around the phantom.

6 Illustrative diagrams of the PPE evaluation results

In the following, two diagrams are given illustrating all evaluated PPEs of the three CT marker configurations (Figure 6) and of the MRI marker configuration (Figure 7).

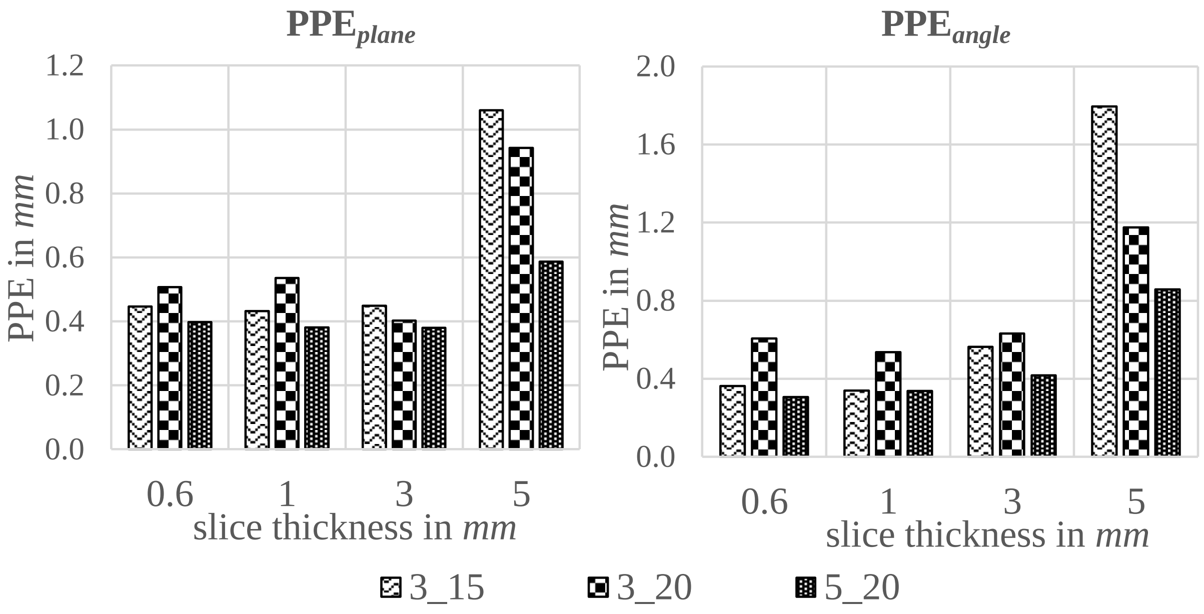

Figure 6: CT measurements: PPE_{plane} and PPE_{angle} depending on the slice thickness and on the marker configuration. Target distance: 100 mm.

Mittmann, B. J., Seitel, A., Echner, G., Johnen, W., Gnirs, R., Maier-Hein, L., Franz, A. M.: **Reattachable fiducial skin marker for automatic multimodality registration**, *Int J Comput Assist Radiol Surg*, Corresponding author: A. M. Franz (alfred.franz@thu.de), Department of Computer Science, Ulm University of Applied Sciences, Albert-Einstein-Allee 55, Ulm, 89081, BW, Germany.

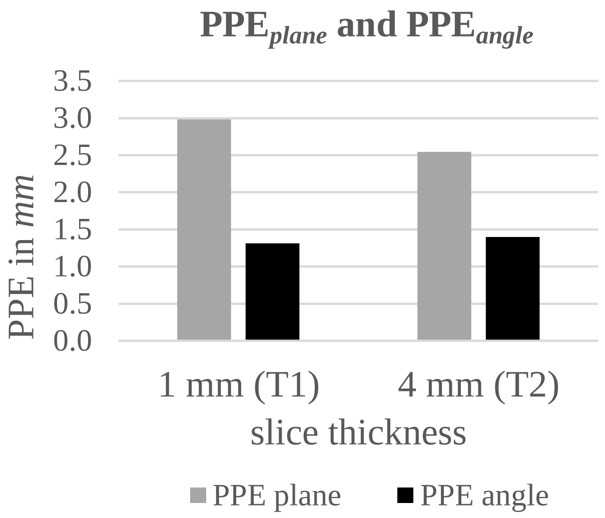

Figure 7: MRI measurements: PPE$_{plane}$ and PPE$_{angle}$ of the 6_20 MRI marker configuration depending on the MRI slice thickness 1 mm (T1-weighted) and 4 mm (T2-weighted). Target distance: 100 mm.

7 Additional remarks regarding the PPE results

As described in the paper, we thoroughly examined the marker localization in a CT and MRI setup and found that the marker could successfully be localized for conventional imaging settings and slice thicknesses. Even though, out of practical reasons, our localization assessment samples the quite high-dimensional space of possible marker poses within the intervention volume only sparsely and does not directly measure the fiducial localization error, the achieved low prediction errors below 0.6 mm (CT) and 1.4 mm (MRI) in the optimal case indicate that accurate localization of the fiducial marker in different imaging modalities can be achieved. At a closer look, we identified the following principal outcomes:

1) The influence of systematic effects of the experimental setup for determining the PPE, such as the repeatability error, the clipping precision and the manufacturing error (< 0.05 mm) of the high precision phantoms, was negligible low compared with the determined PPEs. Thus, the PPEs depended mainly on the error of localizing the fiducial marker.

2) The 5_20 configuration achieved the lowest localization errors in case of CT imaging. Thus, the fiducial marker with the biggest fiducial features and the largest inter-feature distance was the configuration that could be localized most accurately. These results confirmed the findings concerning the theory of the fiducial localization error (FLE) (Maurer et al. 1997) and the TRE published previously by other authors. Nevertheless, it should be examined in a future study, whether other marker configurations varying the number of fiducial features might have a lower localization error than the 5_20 configuration.

3) The localization errors of the MRI measurements were lower for the *angle* phantom than for the *plane* phantom independent of the MRI sequence (T1- or T2-weighted). Compared with the CT measurements, this contrasting behavior was based on the fiducial marker distance to the isocenter, whose position was chosen to coincide approximately with the symmetry point of the *plane* phantom, resulting in a marker distance to the isocenter of about 100 mm for all marker poses of the *plane* phantom, while it was 86 mm $\pm$ 39 mm for the marker poses of the *angle* phantom. As geometric distortions in MRI images are increased with increased distance from the isocenter, these lead to a more inaccurate fiducial marker localization and thus to a greater PPE. Hence, the fiducial marker should be placed on the patient such that it is as close as possible to the isocenter.

4) The MRI localization errors were on average greater compared with the CT measurements. Hence, the MRI fiducial marker could be localized less accurately than the CT adapted fiducial marker. The Beekley *PinPoint©-187* MRI spots used in the fiducial marker were another reason for this observation. Each spot contains a small air bubble

(approx. 15% of total spot volume) producing a void signal in the MRI image. When localizing the fiducial marker, the void signals of the spots' air bubbles might lead to small deviations between the calculated and the real centroids of the fiducial features. This makes the localization of the fiducial marker less accurate and increases the PPE. To eliminate this error, either the localization algorithm should be adapted or, more preferably, MRI capable fiducial features without air bubble should be chosen in the future.

Mittmann, B. J., Seitel, A., Echner, G., Johnen, W., Gnirs, R., Maier-Hein, L., Franz, A. M.: **Reattachable fiducial skin marker for automatic multimodality registration**, *Int J Comput Assist Radiol Surg*, Corresponding author: A. M. Franz (alfred.franz@thu.de), Department of Computer Science, Ulm University of Applied Sciences, Albert-Einstein-Allee 55, Ulm, 89081, BW, Germany.

References

Maurer CR, Fitzpatrick JM, Wang MY, Galloway RL, Maciunas RJ, and Allen GS (1997) Registration of head volume images using implantable fiducial markers. IEEE Transactions on Medical Imaging 16(4): 447–462.

MicronTracker Developer's Manual MTC 2.6, Claron Technology Inc., 2006.

Franz AM, Haidegger T, Birkfellner W, Cleary K, Peters TM, and Maier-Hein L (2014) Electromagnetic Tracking in Medicine – A Review of Technology, Validation, and Applications. IEEE Transactions on Medical Imaging 33(87): 1702–1725.

Nejad-Davarani SP, Kim JP, Du D, and Glide-Hurst C (2019) Large field of view distortion assessment in a low-field MR-linac. Medical Physics 46(5): 2347–2355.
