# Supplementary material for: Reattachable fiducial skin marker for automatic multimodality registration
Source: Int J Comput Assist Radiol Surg. 2022 May 23;17(11):2141–50. doi: 10.1007/s11548-022-02639-7 (PMC9515062; doi:10.1007/s11548-022-02639-7)
Supplement: Supplementary file 3 — (pdf 853 KB) [file 11548_2022_2639_MOESM3_ESM.pdf]

Abdominal phantom study details

In the following sections, we will give (1) detailed information about using the combined modality and accessing its ultrasound (US) images, (2) information regarding the transformation chain $T_{CT \to US}$ and (3) example snapshots of the computed tomography-(CT-)to-US registration.

1) Using the combined modality and accessing its US images

The combined modality (Figure 1) consisted of the small TX1 electromagnetic (EM) field generator (FG) (Polhemus Inc., Colchester, VT, USA) and the curved Telemed US probe (C4.5|50|128Z, Telemed Ltd., Vilnius, Lithuania). It was operated with the Telemed device LogicScan 128. In advance, the TX1 FG has been calibrated by Polhemus with respect to the Telemed curved probe to minimize the tracking error caused by EM field distortions by the probe. For accessing the US images, the US device was connected to the PC running a PlusServer of the PLUS toolkit (plustoolkit.github.io) that accessed the US images and sent them in real time via OpenIGTLink (openigtlink.org) to MITK (mitk.org).

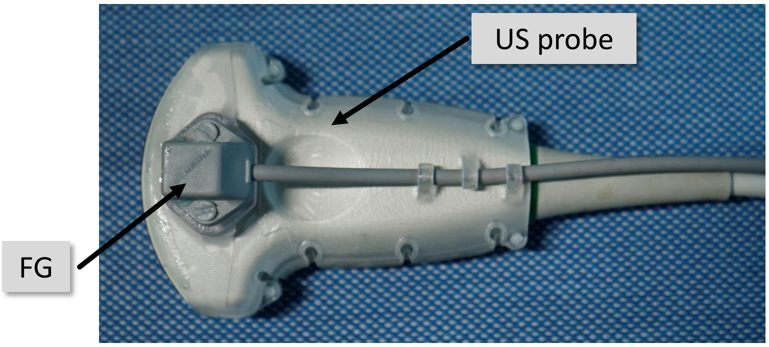

Figure 1: Combined modality used in the abdominal phantom study.

Mittmann, B. J., Seitel, A., Echner, G., Johnen, W., Gnirs, R., Maier-Hein, L., Franz, A. M.: **Reattachable fiducial skin marker for automatic multimodality registration**, *Int J Comput Assist Radiol Surg*, Corresponding author: A. M. Franz (alfred.franz@thu.de), Department of Computer Science, Ulm University of Applied Sciences, Albert-Einstein-Allee 55, Ulm, 89081, BW, Germany.

2) Transformation chain $T_{CT \to US}$

The overall transformation $T_{CT \to US}$ required for registering the CT data to the US image space was calculated by composing the four transformations $T_{FM \to CT}$, $T_{FM \to S}$, $T_{S \to EMT}$ and $T_{US \to EMT}$ as specified in Figure 2 and Equation 1:

$$T_{CT \to US} = T^{-1}_{US \to EMT} \cdot T_{S \to EMT} \cdot T_{FM \to S} \cdot T^{-1}_{FM \to CT} \tag{1}$$

$T^{-1}_{FM \to CT}$ and $T^{-1}_{US \to EMT}$ correspond to the inverse transformations $T_{FM \to CT}$ and $T_{US \to EMT}$, respectively. As the transformations $T_{FM \to S}$ and $T_{US \to EMT}$ were constant during the whole study, they had to be determined only once in advance. $T_{FM \to S}$ could be manually calculated based on the known, constant pose of the fiducial marker coordinate system (CS) relative to the sensor CS, when both the fiducial marker or the sensor holder are clipped on the clipping plate. The *US-to-tracking* transformation ($T_{US \to EMT}$) was determined as described in the paper. Furthermore, the tracking coordinates of the tracked EM sensor corresponded directly to the transformation $T_{S \to EMT}$, whereas the *marker-to-CT* transformation ($T_{FM \to CT}$) was provided by the fiducial marker localization algorithm.

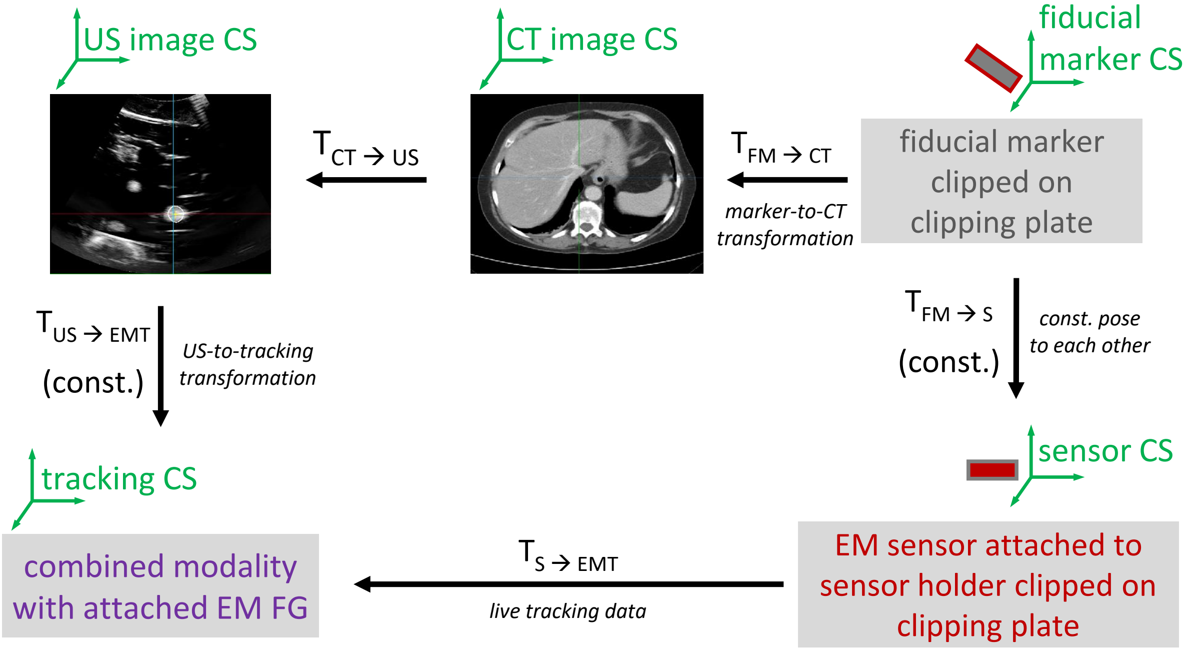

Figure 2: Transformation chain required for registering the CT data to the US image space. Abbreviations: CS = coordinate system; CT = computed tomography; EM = electromagnetic; EMT = electromagnetic tracking; FG = field generator; FM = fiducial marker; S = sensor; US = ultrasound.

3) Example snapshots of the CT-to-US registration

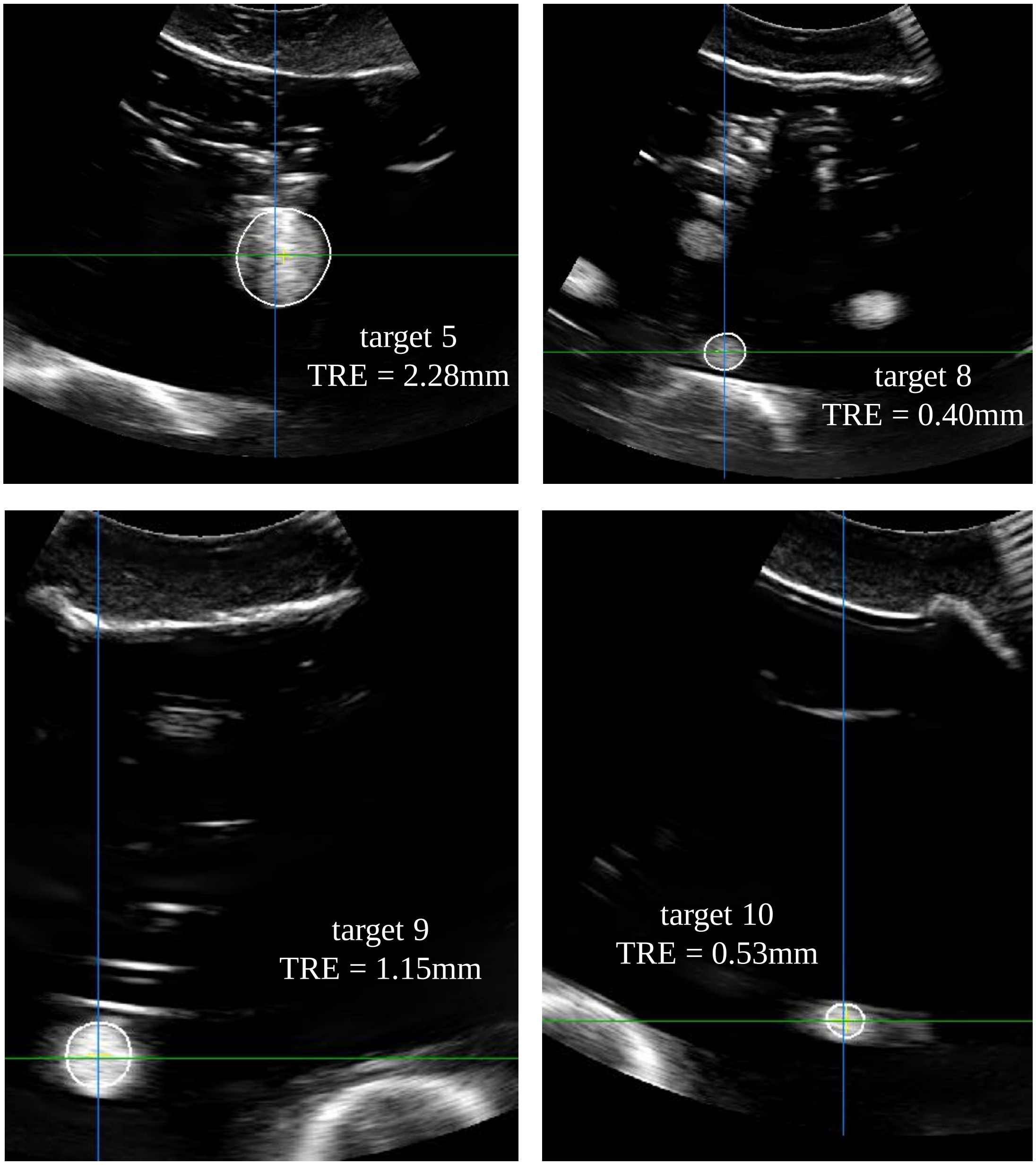

Figure 3: Visual illustration of the resulting CT-to-US registration in case of four different targets of the abdominal phantom and clipping position 1. The pictures show the intersections of the registered target surfaces with the US image plane. The big cross hairs indicate the manually marked centroids of the targets in the US images
