# Supplementary material for: Reattachable fiducial skin marker for automatic multimodality registration
Source: Int J Comput Assist Radiol Surg. 2022 May 23;17(11):2141–50. doi: 10.1007/s11548-022-02639-7 (PMC9515062; doi:10.1007/s11548-022-02639-7)
Supplement: Supplementary file 6 — (pdf 136 KB) [file 11548_2022_2639_MOESM6_ESM.pdf]

Table of contents

\\PHYSIK
E010
Gnirs
BPL_Beekley_Planeboard_Session1_normal
localizer _BPL
localizer _BPL
6_20_1PLANE_T1
6_20_1PLANE_T2
6_20_2PLANE_T1
6_20_2PLANE_T2
6_20_3PLANE_T1
6_20_3PLANE_T2
6_20_4PLANE_T1
6_20_4PLANE_T2
6_20_5PLANE_T1
6_20_5PLANE_T2
6_20_6PLANE_T1
6_20_6PLANE_T2

\\PHYSIK\E010\Gnirs\BPL_Beekley_Planeboard_Session1_normal\localizer _BPL
TA: 0:22 PM: ISO Voxel size: 0.6×0.6×8.0 mmPAT: Off Rel. SNR: 1.00 : fl

Properties

Prio recon	Off
Load images to viewer	On
Inline movie	Off
Auto store images	On
Load images to stamp segments	On
Load images to graphic segments	On
Auto open inline display	Off
Auto close inline display	On
Start measurement without further preparation	On
Wait for user to start	Off
Start measurements	Single measurement

Routine

Slice group	1
Slices	5
Dist. factor	300 %
Position	L0.0 P64.1 H1.4 mm
Orientation	Coronal
Phase enc. dir.	R >> L
Slice group	2
Slices	3
Dist. factor	20 %
Position	L0.0 P64.1 H1.4 mm
Orientation	Sagittal
Phase enc. dir.	A >> P
AutoAlign	---
Phase oversampling	0 %
FoV read	320 mm
FoV phase	100.0 %
Slice thickness	8.0 mm
TR	7.0 ms
TE	2.38 ms
Averages	2
Concatenations	8
Filter	Distortion Corr.(2D), Prescan Normalize, Elliptical filter
Coil elements	BO1-3;SP3,4

Contrast - Common

TR	7.0 ms
TE	2.38 ms
TD	0 ms
MTC	Off
Magn. preparation	None
Flip angle	20 deg
Fat suppr.	None
Water suppr.	None
SWI	Off

Contrast - Dynamic

Averages	2
Averaging mode	Short term
Reconstruction	Magnitude
Measurements	1
Multiple series	Off

Resolution - Common

FoV read	320 mm
FoV phase	100.0 %

Resolution - Common

Slice thickness	8.0 mm
Base resolution	256
Phase resolution	71 %
Phase partial Fourier	Off
Interpolation	On

Resolution - iPAT

PAT mode	None

Resolution - Filter Image

Image Filter	Off
Distortion Corr.	On
Mode	2D
Unfiltered images	Off
Prescan Normalize	On
Unfiltered images	Off
Normalize	Off
B1 filter	Off

Resolution - Filter Rawdata

Raw filter	Off
Elliptical filter	On

Geometry - Common

Slice group	1
Slices	5
Dist. factor	300 %
Position	L0.0 P64.1 H1.4 mm
Orientation	Coronal
Phase enc. dir.	R >> L
Slice group	2
Slices	3
Dist. factor	20 %
Position	L0.0 P64.1 H1.4 mm
Orientation	Sagittal
Phase enc. dir.	A >> P
FoV read	320 mm
FoV phase	100.0 %
Slice thickness	8.0 mm
TR	7.0 ms
Multi-slice mode	Sequential
Series	Interleaved
Concatenations	8

Geometry - AutoAlign

Slice group	1
Position	L0.0 P64.1 H1.4 mm
Orientation	Coronal
Phase enc. dir.	R >> L
Slice group	2
Position	L0.0 P64.1 H1.4 mm
Orientation	Sagittal
Phase enc. dir.	A >> P
AutoAlign	---
Initial Position	L0.0 P64.1 H1.4
L	0.0 mm
P	64.1 mm
H	1.4 mm
Initial Rotation	0.00 deg
Initial Orientation	Coronal

Geometry - Saturation

Saturation mode	Standard
Fat suppr.	None
Water suppr.	None
Special sat.	None

Geometry - Tim Planning Suite

Set-n-Go Protocol	Off
Table position	H
Table position	1 mm
Inline Composing	Off

System - Miscellaneous

Positioning mode	ISO
Table position	H
Table position	1 mm
MSMA	S - C - T
Sagittal	R >> L
Coronal	A >> P
Transversal	H >> F
Coil Combine Mode	Adaptive Combine
Save uncombined	Off
Matrix Optimization	Off
Coil Focus	Flat
AutoAlign	---
Coil Select Mode	Off - AutoCoilSelect

System - Adjustments

B0 Shim mode	Tune up
Adjust with body coil	Off
Confirm freq. adjustment	Off
Assume Dominant Fat	Off
Assume Silicone	Off
Adjustment Tolerance	Auto

System - Adjust Volume

Position	Isocenter
Orientation	Transversal
Rotation	0.00 deg
A >> P	263 mm
R >> L	350 mm
F >> H	350 mm
Reset	Off

System - Tx/Rx

Frequency 1H	63.685812 MHz
Correction factor	1
Gain	High
Img. Scale Cor.	1.000
Reset	Off
? Ref. amplitude 1H	0.000 V

Physio - Signal1

1st Signal/Mode	None
TR	7.0 ms
Concatenations	8
Segments	1

Physio - Cardiac

Magn. preparation	None
Fat suppr.	None
Dark blood	Off
FoV read	320 mm
FoV phase	100.0 %

Physio - Cardiac

Phase resolution	71 %

Physio - PACE

Resp. control	Off
Concatenations	8

Inline - Common

Subtract	Off
Measurements	1
StdDev	Off
Liver registration	Off
Save original images	On

Inline - MIP

MIP-Sag	Off
MIP-Cor	Off
MIP-Tra	Off
MIP-Time	Off
Save original images	On

Inline - Soft Tissue

Wash - In	Off
Wash - Out	Off
TTP	Off
PEI	Off
MIP - time	Off
Measurements	1

Inline - Composing

Inline Composing	Off
Distortion Corr.	On
Mode	2D
Unfiltered images	Off

Inline - MapIt

Save original images	On
MapIt	None
Flip angle	20 deg
Measurements	1
Contrasts	1
TR	7.0 ms
TE	2.38 ms

Sequence - Part 1

Introduction	On
Dimension	2D
Phase stabilisation	Off
Asymmetric echo	Allowed
Contrasts	1
Flow comp.	No
Multi-slice mode	Sequential
Bandwidth	300 Hz/Px

Sequence - Part 2

Segments	1
Acoustic noise reduction	None
RF pulse type	Fast
Gradient mode	Normal
Excitation	Slice-sel.
RF spoiling	On

Sequence - Assistant

Mode	Off

\\PHYSIK\E010\Gnirs\BPL_Beekley_Planeboard_Session1_normal\localizer _BPL

TA: 0:32 PM: ISO Voxel size: 0.6×0.6×8.0 mmPAT: Off Rel. SNR: 1.00 : fl

Properties

Prio recon	Off
Load images to viewer	On
Inline movie	Off
Auto store images	On
Load images to stamp segments	On
Load images to graphic segments	On
Auto open inline display	Off
Auto close inline display	On
Start measurement without further preparation	On
Wait for user to start	Off
Start measurements	Single measurement

Routine

Slice group	1
Slices	9
Dist. factor	200 %
Position	L0.0 P64.1 H1.4 mm
Orientation	Coronal
Phase enc. dir.	R >> L
Slice group	2
Slices	3
Dist. factor	20 %
Position	L0.0 P64.1 H1.4 mm
Orientation	Sagittal
Phase enc. dir.	A >> P
AutoAlign	---
Phase oversampling	0 %
FoV read	320 mm
FoV phase	100.0 %
Slice thickness	8.0 mm
TR	7.0 ms
TE	2.38 ms
Averages	2
Concatenations	12
Filter	Distortion Corr.(2D), Prescan Normalize, Elliptical filter
Coil elements	BO1-3;SP3,4

Contrast - Common

TR	7.0 ms
TE	2.38 ms
TD	0 ms
MTC	Off
Magn. preparation	None
Flip angle	20 deg
Fat suppr.	None
Water suppr.	None
SWI	Off

Contrast - Dynamic

Averages	2
Averaging mode	Short term
Reconstruction	Magnitude
Measurements	1
Multiple series	Off

Resolution - Common

FoV read	320 mm
FoV phase	100.0 %
Slice thickness	8.0 mm
Base resolution	256
Phase resolution	71 %
Phase partial Fourier	Off
Interpolation	On

Resolution - iPAT

PAT mode	None

Resolution - Filter Image

Image Filter	Off
Distortion Corr.	On
Mode	2D
Unfiltered images	Off
Prescan Normalize	On
Unfiltered images	Off
Normalize	Off
B1 filter	Off

Resolution - Filter Rawdata

Raw filter	Off
Elliptical filter	On

Geometry - Common

Slice group	1
Slices	9
Dist. factor	200 %
Position	L0.0 P64.1 H1.4 mm
Orientation	Coronal
Phase enc. dir.	R >> L
Slice group	2
Slices	3
Dist. factor	20 %
Position	L0.0 P64.1 H1.4 mm
Orientation	Sagittal
Phase enc. dir.	A >> P
FoV read	320 mm
FoV phase	100.0 %
Slice thickness	8.0 mm
TR	7.0 ms
Multi-slice mode	Sequential
Series	Interleaved
Concatenations	12

Geometry - AutoAlign

Slice group	1
Position	L0.0 P64.1 H1.4 mm
Orientation	Coronal
Phase enc. dir.	R >> L
Slice group	2
Position	L0.0 P64.1 H1.4 mm
Orientation	Sagittal
Phase enc. dir.	A >> P
AutoAlign	---
Initial Position	L0.0 P64.1 H1.4
L	0.0 mm
P	64.1 mm
H	1.4 mm
Initial Rotation	0.00 deg
Initial Orientation	Coronal

Geometry - Saturation

Saturation mode	Standard
Fat suppr.	None
Water suppr.	None
Special sat.	None

Geometry - Tim Planning Suite

Set-n-Go Protocol	Off
Table position	H
Table position	1 mm
Inline Composing	Off

System - Miscellaneous

Positioning mode	ISO
Table position	H
Table position	1 mm
MSMA	S - C - T
Sagittal	R >> L
Coronal	A >> P
Transversal	H >> F
Coil Combine Mode	Adaptive Combine
Save uncombined	Off
Matrix Optimization	Off
Coil Focus	Flat
AutoAlign	---
Coil Select Mode	Off - AutoCoilSelect

System - Adjustments

B0 Shim mode	Tune up
Adjust with body coil	Off
Confirm freq. adjustment	Off
Assume Dominant Fat	Off
Assume Silicone	Off
Adjustment Tolerance	Auto

System - Adjust Volume

Position	Isocenter
Orientation	Transversal
Rotation	0.00 deg
A >> P	263 mm
R >> L	350 mm
F >> H	350 mm
Reset	Off

System - Tx/Rx

Frequency 1H	63.685812 MHz
Correction factor	1
Gain	High
Img. Scale Cor.	1.000
Reset	Off
? Ref. amplitude 1H	0.000 V

Physio - Signal1

1st Signal/Mode	None
TR	7.0 ms
Concatenations	12
Segments	1

Physio - Cardiac

Magn. preparation	None
Fat suppr.	None
Dark blood	Off
FoV read	320 mm
FoV phase	100.0 %

Physio - Cardiac

Phase resolution	71 %

Physio - PACE

Resp. control	Off
Concatenations	12

Inline - Common

Subtract	Off
Measurements	1
StdDev	Off
Liver registration	Off
Save original images	On

Inline - MIP

MIP-Sag	Off
MIP-Cor	Off
MIP-Tra	Off
MIP-Time	Off
Save original images	On

Inline - Soft Tissue

Wash - In	Off
Wash - Out	Off
TTP	Off
PEI	Off
MIP - time	Off
Measurements	1

Inline - Composing

Inline Composing	Off
Distortion Corr.	On
Mode	2D
Unfiltered images	Off

Inline - MapIt

Save original images	On
MapIt	None
Flip angle	20 deg
Measurements	1
Contrasts	1
TR	7.0 ms
TE	2.38 ms

Sequence - Part 1

Introduction	On
Dimension	2D
Phase stabilisation	Off
Asymmetric echo	Allowed
Contrasts	1
Flow comp.	No
Multi-slice mode	Sequential
Bandwidth	300 Hz/Px

Sequence - Part 2

Segments	1
Acoustic noise reduction	None
RF pulse type	Fast
Gradient mode	Normal
Excitation	Slice-sel.
RF spoiling	On

Sequence - Assistant

Mode	Off

\\PHYSIK\E010\Gnirs\BPL_Beekley_Planeboard_Session1_normal\6_20_1PLANE_T1

TA: 8:12 PM: ISO Voxel size: 0.5×0.5×1.0 mmPAT: 2 Rel. SNR: 1.00 : tfl

Properties

Prio recon	Off
Load images to viewer	On
Inline movie	Off
Auto store images	On
Load images to stamp segments	On
Load images to graphic segments	Off
Auto open inline display	Off
Auto close inline display	On
Start measurement without further preparation	Off
Wait for user to start	Off
Start measurements	Single measurement

Routine

Slab group	1
Slabs	1
Dist. factor	50 %
Position	Isocenter
Orientation	Sagittal
Phase enc. dir.	A >> P
AutoAlign	Head > Brain
Phase oversampling	50 %
Slice oversampling	0.0 %
Slices per slab	320
FoV read	320 mm
FoV phase	100.0 %
Slice thickness	1.00 mm
TR	1880.0 ms
TE	2.63 ms
Averages	1
Concatenations	1
Filter	Distortion Corr.(2D), Prescan Normalize, Elliptical filter
Coil elements	BO1-3;SP3-5

Contrast - Common

TR	1880.0 ms
TE	2.63 ms
Magn. preparation	Non-sel. IR
TI	1030 ms
Flip angle	12 deg
Fat suppr.	None
Water suppr.	None

Contrast - Dynamic

Averages	1
Averaging mode	Long term
Reconstruction	Magnitude
Measurements	1
Multiple series	Off

Resolution - Common

FoV read	320 mm
FoV phase	100.0 %
Slice thickness	1.00 mm
Base resolution	320
Phase resolution	100 %
Slice resolution	80 %
Phase partial Fourier	Off
Slice partial Fourier	Off

Resolution - Common

Interpolation	On

Resolution - iPAT

PAT mode	GRAPPA
Accel. factor PE	2
Ref. lines PE	45
Accel. factor 3D	1
Reference scan mode	Integrated

Resolution - Filter Image

Image Filter	Off
Distortion Corr.	On
Mode	2D
Unfiltered images	Off
Prescan Normalize	On
Unfiltered images	Off
Normalize	Off
B1 filter	Off

Resolution - Filter Rawdata

Raw filter	Off
Elliptical filter	On

Geometry - Common

Slab group	1
Slabs	1
Dist. factor	50 %
Position	Isocenter
Orientation	Sagittal
Phase enc. dir.	A >> P
Slice oversampling	0.0 %
Slices per slab	320
FoV read	320 mm
FoV phase	100.0 %
Slice thickness	1.00 mm
TR	1880.0 ms
Multi-slice mode	Single shot
Series	Ascending
Concatenations	1

Geometry - AutoAlign

Slab group	1
Position	Isocenter
Orientation	Sagittal
Phase enc. dir.	A >> P
AutoAlign	Head > Brain
Initial Position	L0.0 P0.0 H3.0
L	0.0 mm
P	0.0 mm
H	3.0 mm
Initial Rotation	-0.01 deg
Initial Orientation	Sagittal

Geometry - Navigator

Geometry - Tim Planning Suite

Set-n-Go Protocol	Off
Table position	H
Table position	3 mm
Inline Composing	Off

System - Miscellaneous

Positioning mode	ISO
Table position	H
Table position	3 mm
MSMA	S - C - T
Sagittal	R >> L
Coronal	A >> P
Transversal	F >> H
Coil Combine Mode	Adaptive Combine
Save uncombined	Off
Matrix Optimization	Off
Coil Focus	Flat
AutoAlign	Head > Brain
Coil Select Mode	Default

System - Adjustments

B0 Shim mode	Tune up
Adjust with body coil	Off
Confirm freq. adjustment	Off
Assume Dominant Fat	Off
Assume Silicone	Off
Adjustment Tolerance	Auto

System - Adjust Volume

Position	Isocenter
Orientation	Transversal
Rotation	0.00 deg
A >> P	263 mm
R >> L	350 mm
F >> H	350 mm
Reset	Off

System - Tx/Rx

Frequency 1H	63.685812 MHz
Correction factor	1
Gain	Low
Img. Scale Cor.	1.000
Reset	Off
? Ref. amplitude 1H	0.000 V

Physio - Signal1

1st Signal/Mode	None
TR	1880.0 ms
Concatenations	1

Physio - Cardiac

Magn. preparation	Non-sel. IR
TI	1030 ms
Fat suppr.	None
Dark blood	Off
FoV read	320 mm
FoV phase	100.0 %
Phase resolution	100 %

Physio - PACE

Resp. control	Off
Concatenations	1

Inline - Common

Subtract	On
Save images	On
Autoscaling	Off
Scaling factor	1
Offset	0

Inline - Common

Subtrahend	1
Subtraction group	1
Measurements	1
StdDev	Off
Save original images	On

Inline - MIP

MIP-Sag	Off
MIP-Cor	Off
MIP-Tra	Off
MIP-Time	Off
Save original images	On

Inline - Composing

Inline Composing	Off
Distortion Corr.	On
Mode	2D
Unfiltered images	Off

Inline - MapIt

Save original images	On
MapIt	None
Flip angle	12 deg
Measurements	1
TR	1880.0 ms
TE	2.63 ms

Sequence - Part 1

Introduction	On
Dimension	3D
Elliptical scanning	Off
Reordering	Linear
Asymmetric echo	Allowed
Flow comp.	No
Multi-slice mode	Single shot
Echo spacing	6.6 ms
Bandwidth	200 Hz/Px

Sequence - Part 2

RF pulse type	Fast
Gradient mode	Fast
Excitation	Slab-sel.
RF spoiling	On
Incr. Gradient spoiling	Off
Turbo factor	256

Sequence - Assistant

Mode	Off

\\PHYSIK\E010\Gnirs\BPL_Beekley_Planeboard_Session1_normal\6_20_1PLANE_T2
TA: 9:16 PM: FIX Voxel size: 0.4×0.4×4.0 mmPAT: 2 Rel. SNR: 1.00 : tseR

Properties

Prio recon	Off
Load images to viewer	On
Inline movie	Off
Auto store images	On
Load images to stamp segments	On
Load images to graphic segments	Off
Auto open inline display	Off
Auto close inline display	Off
Start measurement without further preparation	Off
Wait for user to start	Off
Start measurements	Single measurement

Routine

Slice group	1
Slices	80
Dist. factor	0 %
Position	Isocenter
Orientation	Sagittal
Phase enc. dir.	H >> F
AutoAlign	Head > Brain
Phase oversampling	0 %
FoV read	320 mm
FoV phase	100.0 %
Slice thickness	4.0 mm
TR	6450.0 ms
TE	107.0 ms
Averages	3
Concatenations	2
Filter	Distortion Corr.(2D), Prescan Normalize, Elliptical filter
Coil elements	BO1-3;SP3-5

Contrast - Common

TR	6450.0 ms
TE	107.0 ms
TD	0.0 ms
MTC	Off
Magn. preparation	None
Flip angle	180 deg
Fat suppr.	None
Water suppr.	None
Restore magn.	On

Contrast - Dynamic

Averages	3
Averaging mode	Short term
Reconstruction	Magnitude
Measurements	1
Multiple series	Each measurement

Resolution - Common

FoV read	320 mm
FoV phase	100.0 %
Slice thickness	4.0 mm
Base resolution	448
Phase resolution	90 %
Phase partial Fourier	Off
Trajectory	Cartesian
Interpolation	On

Resolution - iPAT

PAT mode	GRAPPA
Accel. factor PE	2
Ref. lines PE	32
Reference scan mode	Self-calibration

Resolution - Filter Image

Image Filter	Off
Distortion Corr.	On
Mode	2D
Unfiltered images	Off
Prescan Normalize	On
Unfiltered images	Off
Normalize	Off
B1 filter	Off

Resolution - Filter Rawdata

Raw filter	Off
Elliptical filter	On

Geometry - Common

Slice group	1
Slices	80
Dist. factor	0 %
Position	Isocenter
Orientation	Sagittal
Phase enc. dir.	H >> F
FoV read	320 mm
FoV phase	100.0 %
Slice thickness	4.0 mm
TR	6450.0 ms
Multi-slice mode	Interleaved
Series	Interleaved
Concatenations	2

Geometry - AutoAlign

Slice group	1
Position	Isocenter
Orientation	Sagittal
Phase enc. dir.	H >> F
AutoAlign	Head > Brain
Initial Position	Isocenter
L	0.0 mm
P	0.0 mm
H	0.0 mm
Initial Rotation	89.99 deg
Initial Orientation	Sagittal

Geometry - Saturation

Fat suppr.	None
Water suppr.	None
Restore magn.	On
Special sat.	None

Geometry - Navigator

Geometry - Tim Planning Suite

Set-n-Go Protocol	Off
Table position	H
Table position	3 mm
Inline Composing	Off

System - Miscellaneous

Positioning mode	FIX
Table position	H
Table position	3 mm
MSMA	S - C - T
Sagittal	R >> L
Coronal	A >> P
Transversal	F >> H
Coil Combine Mode	Adaptive Combine
Save uncombined	Off
Matrix Optimization	Off
Coil Focus	Flat
AutoAlign	Head > Brain
Coil Select Mode	Default

System - Adjustments

B0 Shim mode	Tune up
Adjust with body coil	Off
Confirm freq. adjustment	Off
Assume Dominant Fat	Off
Assume Silicone	Off
Adjustment Tolerance	Auto

System - Adjust Volume

Position	Isocenter
Orientation	Transversal
Rotation	0.00 deg
A >> P	263 mm
R >> L	350 mm
F >> H	350 mm
Reset	Off

System - Tx/Rx

Frequency 1H	63.685812 MHz
Correction factor	1
Gain	High
Img. Scale Cor.	1.000
Reset	Off
? Ref. amplitude 1H	0.000 V

Physio - Signal1

1st Signal/Mode	None
TR	6450.0 ms
Concatenations	2

Physio - Cardiac

Magn. preparation	None
Fat suppr.	None
Dark blood	Off
FoV read	320 mm
FoV phase	100.0 %
Phase resolution	90 %
Trajectory	Cartesian

Physio - PACE

Resp. control	Off
Concatenations	2

Inline - Common

Subtract	Off
Measurements	1
StdDev	Off
Save original images	On

Inline - MIP

MIP-Sag	Off
MIP-Cor	Off
MIP-Tra	Off
MIP-Time	Off
Save original images	On

Inline - Composing

Inline Composing	Off
Distortion Corr.	On
Mode	2D
Unfiltered images	Off

Sequence - Part 1

Introduction	On
Dimension	2D
Compensate T2 decay	Off
Reduce Motion Sens.	On
Contrasts	1
Flow comp.	No
Multi-slice mode	Interleaved
Free echo spacing	Off
Echo spacing	9.74 ms
Bandwidth	199 Hz/Px

Sequence - Part 2

Define	Turbo factor
Echo trains per slice	14
Phase correction	Automatic
Acoustic noise reduction	None
RF pulse type	Normal
Gradient mode	Normal
WARP	Off
Red. EC sensitivity	Off
Turbo factor	15

Sequence - Assistant

Mode	Off
Allowed delay	30 s

\\PHYSIK\E010\Gnirs\BPL_Beekley_Planeboard_Session1_normal\6_20_2PLANE_T1
TA: 8:12 PM: ISO Voxel size: 0.5×0.5×1.0 mmPAT: 2 Rel. SNR: 1.00 : tfl

Properties

Prio recon	Off
Load images to viewer	On
Inline movie	Off
Auto store images	On
Load images to stamp segments	On
Load images to graphic segments	Off
Auto open inline display	Off
Auto close inline display	On
Start measurement without further preparation	Off
Wait for user to start	Off
Start measurements	Single measurement

Routine

Slab group	1
Slabs	1
Dist. factor	50 %
Position	Isocenter
Orientation	Sagittal
Phase enc. dir.	A >> P
AutoAlign	Head > Brain
Phase oversampling	50 %
Slice oversampling	0.0 %
Slices per slab	320
FoV read	320 mm
FoV phase	100.0 %
Slice thickness	1.00 mm
TR	1880.0 ms
TE	2.63 ms
Averages	1
Concatenations	1
Filter	Distortion Corr.(2D), Prescan Normalize, Elliptical filter
Coil elements	BO1-3;SP3-5

Contrast - Common

TR	1880.0 ms
TE	2.63 ms
Magn. preparation	Non-sel. IR
TI	1030 ms
Flip angle	12 deg
Fat suppr.	None
Water suppr.	None

Contrast - Dynamic

Averages	1
Averaging mode	Long term
Reconstruction	Magnitude
Measurements	1
Multiple series	Off

Resolution - Common

FoV read	320 mm
FoV phase	100.0 %
Slice thickness	1.00 mm
Base resolution	320
Phase resolution	100 %
Slice resolution	80 %
Phase partial Fourier	Off
Slice partial Fourier	Off

Resolution - Common

Interpolation	On

Resolution - iPAT

PAT mode	GRAPPA
Accel. factor PE	2
Ref. lines PE	45
Accel. factor 3D	1
Reference scan mode	Integrated

Resolution - Filter Image

Image Filter	Off
Distortion Corr.	On
Mode	2D
Unfiltered images	Off
Prescan Normalize	On
Unfiltered images	Off
Normalize	Off
B1 filter	Off

Resolution - Filter Rawdata

Raw filter	Off
Elliptical filter	On

Geometry - Common

Slab group	1
Slabs	1
Dist. factor	50 %
Position	Isocenter
Orientation	Sagittal
Phase enc. dir.	A >> P
Slice oversampling	0.0 %
Slices per slab	320
FoV read	320 mm
FoV phase	100.0 %
Slice thickness	1.00 mm
TR	1880.0 ms
Multi-slice mode	Single shot
Series	Ascending
Concatenations	1

Geometry - AutoAlign

Slab group	1
Position	Isocenter
Orientation	Sagittal
Phase enc. dir.	A >> P
AutoAlign	Head > Brain
Initial Position	L0.0 P0.0 H3.0
L	0.0 mm
P	0.0 mm
H	3.0 mm
Initial Rotation	-0.01 deg
Initial Orientation	Sagittal

Geometry - Navigator

Geometry - Tim Planning Suite

Set-n-Go Protocol	Off
Table position	H
Table position	3 mm
Inline Composing	Off

System - Miscellaneous

Positioning mode	ISO
Table position	H
Table position	3 mm
MSMA	S - C - T
Sagittal	R >> L
Coronal	A >> P
Transversal	F >> H
Coil Combine Mode	Adaptive Combine
Save uncombined	Off
Matrix Optimization	Off
Coil Focus	Flat
AutoAlign	Head > Brain
Coil Select Mode	Default

System - Adjustments

B0 Shim mode	Tune up
Adjust with body coil	Off
Confirm freq. adjustment	Off
Assume Dominant Fat	Off
Assume Silicone	Off
Adjustment Tolerance	Auto

System - Adjust Volume

Position	Isocenter
Orientation	Transversal
Rotation	0.00 deg
A >> P	263 mm
R >> L	350 mm
F >> H	350 mm
Reset	Off

System - Tx/Rx

Frequency 1H	63.685812 MHz
Correction factor	1
Gain	Low
Img. Scale Cor.	1.000
Reset	Off
? Ref. amplitude 1H	0.000 V

Physio - Signal1

1st Signal/Mode	None
TR	1880.0 ms
Concatenations	1

Physio - Cardiac

Magn. preparation	Non-sel. IR
TI	1030 ms
Fat suppr.	None
Dark blood	Off
FoV read	320 mm
FoV phase	100.0 %
Phase resolution	100 %

Physio - PACE

Resp. control	Off
Concatenations	1

Inline - Common

Subtract	On
Save images	On
Autoscaling	Off
Scaling factor	1
Offset	0

Inline - Common

Subtrahend	1
Subtraction group	1
Measurements	1
StdDev	Off
Save original images	On

Inline - MIP

MIP-Sag	Off
MIP-Cor	Off
MIP-Tra	Off
MIP-Time	Off
Save original images	On

Inline - Composing

Inline Composing	Off
Distortion Corr.	On
Mode	2D
Unfiltered images	Off

Inline - MapIt

Save original images	On
MapIt	None
Flip angle	12 deg
Measurements	1
TR	1880.0 ms
TE	2.63 ms

Sequence - Part 1

Introduction	On
Dimension	3D
Elliptical scanning	Off
Reordering	Linear
Asymmetric echo	Allowed
Flow comp.	No
Multi-slice mode	Single shot
Echo spacing	6.6 ms
Bandwidth	200 Hz/Px

Sequence - Part 2

RF pulse type	Fast
Gradient mode	Fast
Excitation	Slab-sel.
RF spoiling	On
Incr. Gradient spoiling	Off
Turbo factor	256

Sequence - Assistant

Mode	Off

\\PHYSIK\E010\Gnirs\BPL_Beekley_Planeboard_Session1_normal\6_20_2PLANE_T2
TA: 9:16 PM: FIX Voxel size: 0.4×0.4×4.0 mmPAT: 2 Rel. SNR: 1.00 : tseR

Properties

Prio recon	Off
Load images to viewer	On
Inline movie	Off
Auto store images	On
Load images to stamp segments	On
Load images to graphic segments	Off
Auto open inline display	Off
Auto close inline display	Off
Start measurement without further preparation	Off
Wait for user to start	Off
Start measurements	Single measurement

Routine

Slice group	1
Slices	80
Dist. factor	0 %
Position	Isocenter
Orientation	Sagittal
Phase enc. dir.	H >> F
AutoAlign	Head > Brain
Phase oversampling	0 %
FoV read	320 mm
FoV phase	100.0 %
Slice thickness	4.0 mm
TR	6450.0 ms
TE	107.0 ms
Averages	3
Concatenations	2
Filter	Distortion Corr.(2D), Prescan Normalize, Elliptical filter
Coil elements	BO1-3;SP3-5

Contrast - Common

TR	6450.0 ms
TE	107.0 ms
TD	0.0 ms
MTC	Off
Magn. preparation	None
Flip angle	180 deg
Fat suppr.	None
Water suppr.	None
Restore magn.	On

Contrast - Dynamic

Averages	3
Averaging mode	Short term
Reconstruction	Magnitude
Measurements	1
Multiple series	Each measurement

Resolution - Common

FoV read	320 mm
FoV phase	100.0 %
Slice thickness	4.0 mm
Base resolution	448
Phase resolution	90 %
Phase partial Fourier	Off
Trajectory	Cartesian
Interpolation	On

Resolution - iPAT

PAT mode	GRAPPA
Accel. factor PE	2
Ref. lines PE	32
Reference scan mode	Self-calibration

Resolution - Filter Image

Image Filter	Off
Distortion Corr.	On
Mode	2D
Unfiltered images	Off
Prescan Normalize	On
Unfiltered images	Off
Normalize	Off
B1 filter	Off

Resolution - Filter Rawdata

Raw filter	Off
Elliptical filter	On

Geometry - Common

Slice group	1
Slices	80
Dist. factor	0 %
Position	Isocenter
Orientation	Sagittal
Phase enc. dir.	H >> F
FoV read	320 mm
FoV phase	100.0 %
Slice thickness	4.0 mm
TR	6450.0 ms
Multi-slice mode	Interleaved
Series	Interleaved
Concatenations	2

Geometry - AutoAlign

Slice group	1
Position	Isocenter
Orientation	Sagittal
Phase enc. dir.	H >> F
AutoAlign	Head > Brain
Initial Position	Isocenter
L	0.0 mm
P	0.0 mm
H	0.0 mm
Initial Rotation	89.99 deg
Initial Orientation	Sagittal

Geometry - Saturation

Fat suppr.	None
Water suppr.	None
Restore magn.	On
Special sat.	None

Geometry - Navigator

Geometry - Tim Planning Suite

Set-n-Go Protocol	Off
Table position	H
Table position	3 mm
Inline Composing	Off

System - Miscellaneous

Positioning mode	FIX
Table position	H
Table position	3 mm
MSMA	S - C - T
Sagittal	R >> L
Coronal	A >> P
Transversal	F >> H
Coil Combine Mode	Adaptive Combine
Save uncombined	Off
Matrix Optimization	Off
Coil Focus	Flat
AutoAlign	Head > Brain
Coil Select Mode	Default

System - Adjustments

B0 Shim mode	Tune up
Adjust with body coil	Off
Confirm freq. adjustment	Off
Assume Dominant Fat	Off
Assume Silicone	Off
Adjustment Tolerance	Auto

System - Adjust Volume

Position	Isocenter
Orientation	Transversal
Rotation	0.00 deg
A >> P	263 mm
R >> L	350 mm
F >> H	350 mm
Reset	Off

System - Tx/Rx

Frequency 1H	63.685812 MHz
Correction factor	1
Gain	High
Img. Scale Cor.	1.000
Reset	Off
? Ref. amplitude 1H	0.000 V

Physio - Signal1

1st Signal/Mode	None
TR	6450.0 ms
Concatenations	2

Physio - Cardiac

Magn. preparation	None
Fat suppr.	None
Dark blood	Off
FoV read	320 mm
FoV phase	100.0 %
Phase resolution	90 %
Trajectory	Cartesian

Physio - PACE

Resp. control	Off
Concatenations	2

Inline - Common

Subtract	Off
Measurements	1
StdDev	Off
Save original images	On

Inline - MIP

MIP-Sag	Off
MIP-Cor	Off
MIP-Tra	Off
MIP-Time	Off
Save original images	On

Inline - Composing

Inline Composing	Off
Distortion Corr.	On
Mode	2D
Unfiltered images	Off

Sequence - Part 1

Introduction	On
Dimension	2D
Compensate T2 decay	Off
Reduce Motion Sens.	On
Contrasts	1
Flow comp.	No
Multi-slice mode	Interleaved
Free echo spacing	Off
Echo spacing	9.74 ms
Bandwidth	199 Hz/Px

Sequence - Part 2

Define	Turbo factor
Echo trains per slice	14
Phase correction	Automatic
Acoustic noise reduction	None
RF pulse type	Normal
Gradient mode	Normal
WARP	Off
Red. EC sensitivity	Off
Turbo factor	15

Sequence - Assistant

Mode	Off
Allowed delay	30 s

\\PHYSIK\E010\Gnirs\BPL_Beekley_Planeboard_Session1_normal\6_20_3PLANE_T1
TA: 8:12 PM: ISO Voxel size: 0.5×0.5×1.0 mmPAT: 2 Rel. SNR: 1.00 : tfl

Properties

Prio recon	Off
Load images to viewer	On
Inline movie	Off
Auto store images	On
Load images to stamp segments	On
Load images to graphic segments	Off
Auto open inline display	Off
Auto close inline display	On
Start measurement without further preparation	Off
Wait for user to start	Off
Start measurements	Single measurement

Routine

Slab group	1
Slabs	1
Dist. factor	50 %
Position	Isocenter
Orientation	Sagittal
Phase enc. dir.	A >> P
AutoAlign	Head > Brain
Phase oversampling	50 %
Slice oversampling	0.0 %
Slices per slab	320
FoV read	320 mm
FoV phase	100.0 %
Slice thickness	1.00 mm
TR	1880.0 ms
TE	2.63 ms
Averages	1
Concatenations	1
Filter	Distortion Corr.(2D), Prescan Normalize, Elliptical filter
Coil elements	BO1-3;SP3-5

Contrast - Common

TR	1880.0 ms
TE	2.63 ms
Magn. preparation	Non-sel. IR
TI	1030 ms
Flip angle	12 deg
Fat suppr.	None
Water suppr.	None

Contrast - Dynamic

Averages	1
Averaging mode	Long term
Reconstruction	Magnitude
Measurements	1
Multiple series	Off

Resolution - Common

FoV read	320 mm
FoV phase	100.0 %
Slice thickness	1.00 mm
Base resolution	320
Phase resolution	100 %
Slice resolution	80 %
Phase partial Fourier	Off
Slice partial Fourier	Off

Resolution - Common

Interpolation	On

Resolution - iPAT

PAT mode	GRAPPA
Accel. factor PE	2
Ref. lines PE	45
Accel. factor 3D	1
Reference scan mode	Integrated

Resolution - Filter Image

Image Filter	Off
Distortion Corr.	On
Mode	2D
Unfiltered images	Off
Prescan Normalize	On
Unfiltered images	Off
Normalize	Off
B1 filter	Off

Resolution - Filter Rawdata

Raw filter	Off
Elliptical filter	On

Geometry - Common

Slab group	1
Slabs	1
Dist. factor	50 %
Position	Isocenter
Orientation	Sagittal
Phase enc. dir.	A >> P
Slice oversampling	0.0 %
Slices per slab	320
FoV read	320 mm
FoV phase	100.0 %
Slice thickness	1.00 mm
TR	1880.0 ms
Multi-slice mode	Single shot
Series	Ascending
Concatenations	1

Geometry - AutoAlign

Slab group	1
Position	Isocenter
Orientation	Sagittal
Phase enc. dir.	A >> P
AutoAlign	Head > Brain
Initial Position	L0.0 P0.0 H3.0
L	0.0 mm
P	0.0 mm
H	3.0 mm
Initial Rotation	-0.01 deg
Initial Orientation	Sagittal

Geometry - Navigator

Geometry - Tim Planning Suite

Set-n-Go Protocol	Off
Table position	H
Table position	3 mm
Inline Composing	Off

System - Miscellaneous

Positioning mode	ISO
Table position	H
Table position	3 mm
MSMA	S - C - T
Sagittal	R >> L
Coronal	A >> P
Transversal	F >> H
Coil Combine Mode	Adaptive Combine
Save uncombined	Off
Matrix Optimization	Off
Coil Focus	Flat
AutoAlign	Head > Brain
Coil Select Mode	Default

System - Adjustments

B0 Shim mode	Tune up
Adjust with body coil	Off
Confirm freq. adjustment	Off
Assume Dominant Fat	Off
Assume Silicone	Off
Adjustment Tolerance	Auto

System - Adjust Volume

Position	Isocenter
Orientation	Transversal
Rotation	0.00 deg
A >> P	263 mm
R >> L	350 mm
F >> H	350 mm
Reset	Off

System - Tx/Rx

Frequency 1H	63.685812 MHz
Correction factor	1
Gain	Low
Img. Scale Cor.	1.000
Reset	Off
? Ref. amplitude 1H	0.000 V

Physio - Signal1

1st Signal/Mode	None
TR	1880.0 ms
Concatenations	1

Physio - Cardiac

Magn. preparation	Non-sel. IR
TI	1030 ms
Fat suppr.	None
Dark blood	Off
FoV read	320 mm
FoV phase	100.0 %
Phase resolution	100 %

Physio - PACE

Resp. control	Off
Concatenations	1

Inline - Common

Subtract	On
Save images	On
Autoscaling	Off
Scaling factor	1
Offset	0

Inline - Common

Subtrahend	1
Subtraction group	1
Measurements	1
StdDev	Off
Save original images	On

Inline - MIP

MIP-Sag	Off
MIP-Cor	Off
MIP-Tra	Off
MIP-Time	Off
Save original images	On

Inline - Composing

Inline Composing	Off
Distortion Corr.	On
Mode	2D
Unfiltered images	Off

Inline - MapIt

Save original images	On
MapIt	None
Flip angle	12 deg
Measurements	1
TR	1880.0 ms
TE	2.63 ms

Sequence - Part 1

Introduction	On
Dimension	3D
Elliptical scanning	Off
Reordering	Linear
Asymmetric echo	Allowed
Flow comp.	No
Multi-slice mode	Single shot
Echo spacing	6.6 ms
Bandwidth	200 Hz/Px

Sequence - Part 2

RF pulse type	Fast
Gradient mode	Fast
Excitation	Slab-sel.
RF spoiling	On
Incr. Gradient spoiling	Off
Turbo factor	256

Sequence - Assistant

Mode	Off

\\PHYSIK\E010\Gnirs\BPL_Beekley_Planeboard_Session1_normal\6_20_3PLANE_T2
TA: 9:16 PM: FIX Voxel size: 0.4×0.4×4.0 mmPAT: 2 Rel. SNR: 1.00 : tseR

Properties

Prio recon	Off
Load images to viewer	On
Inline movie	Off
Auto store images	On
Load images to stamp segments	On
Load images to graphic segments	Off
Auto open inline display	Off
Auto close inline display	Off
Start measurement without further preparation	Off
Wait for user to start	Off
Start measurements	Single measurement

Routine

Slice group	1
Slices	80
Dist. factor	0 %
Position	Isocenter
Orientation	Sagittal
Phase enc. dir.	H >> F
AutoAlign	Head > Brain
Phase oversampling	0 %
FoV read	320 mm
FoV phase	100.0 %
Slice thickness	4.0 mm
TR	6450.0 ms
TE	107.0 ms
Averages	3
Concatenations	2
Filter	Distortion Corr.(2D), Prescan Normalize, Elliptical filter
Coil elements	BO1-3;SP3-5

Contrast - Common

TR	6450.0 ms
TE	107.0 ms
TD	0.0 ms
MTC	Off
Magn. preparation	None
Flip angle	180 deg
Fat suppr.	None
Water suppr.	None
Restore magn.	On

Contrast - Dynamic

Averages	3
Averaging mode	Short term
Reconstruction	Magnitude
Measurements	1
Multiple series	Each measurement

Resolution - Common

FoV read	320 mm
FoV phase	100.0 %
Slice thickness	4.0 mm
Base resolution	448
Phase resolution	90 %
Phase partial Fourier	Off
Trajectory	Cartesian
Interpolation	On

Resolution - iPAT

PAT mode	GRAPPA
Accel. factor PE	2
Ref. lines PE	32
Reference scan mode	Self-calibration

Resolution - Filter Image

Image Filter	Off
Distortion Corr.	On
Mode	2D
Unfiltered images	Off
Prescan Normalize	On
Unfiltered images	Off
Normalize	Off
B1 filter	Off

Resolution - Filter Rawdata

Raw filter	Off
Elliptical filter	On

Geometry - Common

Slice group	1
Slices	80
Dist. factor	0 %
Position	Isocenter
Orientation	Sagittal
Phase enc. dir.	H >> F
FoV read	320 mm
FoV phase	100.0 %
Slice thickness	4.0 mm
TR	6450.0 ms
Multi-slice mode	Interleaved
Series	Interleaved
Concatenations	2

Geometry - AutoAlign

Slice group	1
Position	Isocenter
Orientation	Sagittal
Phase enc. dir.	H >> F
AutoAlign	Head > Brain
Initial Position	Isocenter
L	0.0 mm
P	0.0 mm
H	0.0 mm
Initial Rotation	89.99 deg
Initial Orientation	Sagittal

Geometry - Saturation

Fat suppr.	None
Water suppr.	None
Restore magn.	On
Special sat.	None

Geometry - Navigator

Geometry - Tim Planning Suite

Set-n-Go Protocol	Off
Table position	H
Table position	3 mm
Inline Composing	Off

System - Miscellaneous

Positioning mode	FIX
Table position	H
Table position	3 mm
MSMA	S - C - T
Sagittal	R >> L
Coronal	A >> P
Transversal	F >> H
Coil Combine Mode	Adaptive Combine
Save uncombined	Off
Matrix Optimization	Off
Coil Focus	Flat
AutoAlign	Head > Brain
Coil Select Mode	Default

System - Adjustments

B0 Shim mode	Tune up
Adjust with body coil	Off
Confirm freq. adjustment	Off
Assume Dominant Fat	Off
Assume Silicone	Off
Adjustment Tolerance	Auto

System - Adjust Volume

Position	Isocenter
Orientation	Transversal
Rotation	0.00 deg
A >> P	263 mm
R >> L	350 mm
F >> H	350 mm
Reset	Off

System - Tx/Rx

Frequency 1H	63.685812 MHz
Correction factor	1
Gain	High
Img. Scale Cor.	1.000
Reset	Off
? Ref. amplitude 1H	0.000 V

Physio - Signal1

1st Signal/Mode	None
TR	6450.0 ms
Concatenations	2

Physio - Cardiac

Magn. preparation	None
Fat suppr.	None
Dark blood	Off
FoV read	320 mm
FoV phase	100.0 %
Phase resolution	90 %
Trajectory	Cartesian

Physio - PACE

Resp. control	Off
Concatenations	2

Inline - Common

Subtract	Off
Measurements	1
StdDev	Off
Save original images	On

Inline - MIP

MIP-Sag	Off
MIP-Cor	Off
MIP-Tra	Off
MIP-Time	Off
Save original images	On

Inline - Composing

Inline Composing	Off
Distortion Corr.	On
Mode	2D
Unfiltered images	Off

Sequence - Part 1

Introduction	On
Dimension	2D
Compensate T2 decay	Off
Reduce Motion Sens.	On
Contrasts	1
Flow comp.	No
Multi-slice mode	Interleaved
Free echo spacing	Off
Echo spacing	9.74 ms
Bandwidth	199 Hz/Px

Sequence - Part 2

Define	Turbo factor
Echo trains per slice	14
Phase correction	Automatic
Acoustic noise reduction	None
RF pulse type	Normal
Gradient mode	Normal
WARP	Off
Red. EC sensitivity	Off
Turbo factor	15

Sequence - Assistant

Mode	Off
Allowed delay	30 s

\\PHYSIK\E010\Gnirs\BPL_Beekley_Planeboard_Session1_normal\6_20_4PLANE_T1

TA: 8:12 PM: ISO Voxel size: 0.5×0.5×1.0 mmPAT: 2 Rel. SNR: 1.00 : tfl

Properties

Prio recon	Off
Load images to viewer	On
Inline movie	Off
Auto store images	On
Load images to stamp segments	On
Load images to graphic segments	Off
Auto open inline display	Off
Auto close inline display	On
Start measurement without further preparation	Off
Wait for user to start	Off
Start measurements	Single measurement

Routine

Slab group	1
Slabs	1
Dist. factor	50 %
Position	Isocenter
Orientation	Sagittal
Phase enc. dir.	A >> P
AutoAlign	Head > Brain
Phase oversampling	50 %
Slice oversampling	0.0 %
Slices per slab	320
FoV read	320 mm
FoV phase	100.0 %
Slice thickness	1.00 mm
TR	1880.0 ms
TE	2.63 ms
Averages	1
Concatenations	1
Filter	Distortion Corr.(2D), Prescan Normalize, Elliptical filter
Coil elements	BO1-3;SP3-5

Contrast - Common

TR	1880.0 ms
TE	2.63 ms
Magn. preparation	Non-sel. IR
TI	1030 ms
Flip angle	12 deg
Fat suppr.	None
Water suppr.	None

Contrast - Dynamic

Averages	1
Averaging mode	Long term
Reconstruction	Magnitude
Measurements	1
Multiple series	Off

Resolution - Common

FoV read	320 mm
FoV phase	100.0 %
Slice thickness	1.00 mm
Base resolution	320
Phase resolution	100 %
Slice resolution	80 %
Phase partial Fourier	Off
Slice partial Fourier	Off

Resolution - Common

Interpolation	On

Resolution - iPAT

PAT mode	GRAPPA
Accel. factor PE	2
Ref. lines PE	45
Accel. factor 3D	1
Reference scan mode	Integrated

Resolution - Filter Image

Image Filter	Off
Distortion Corr.	On
Mode	2D
Unfiltered images	Off
Prescan Normalize	On
Unfiltered images	Off
Normalize	Off
B1 filter	Off

Resolution - Filter Rawdata

Raw filter	Off
Elliptical filter	On

Geometry - Common

Slab group	1
Slabs	1
Dist. factor	50 %
Position	Isocenter
Orientation	Sagittal
Phase enc. dir.	A >> P
Slice oversampling	0.0 %
Slices per slab	320
FoV read	320 mm
FoV phase	100.0 %
Slice thickness	1.00 mm
TR	1880.0 ms
Multi-slice mode	Single shot
Series	Ascending
Concatenations	1

Geometry - AutoAlign

Slab group	1
Position	Isocenter
Orientation	Sagittal
Phase enc. dir.	A >> P
AutoAlign	Head > Brain
Initial Position	L0.0 P0.0 H3.0
L	0.0 mm
P	0.0 mm
H	3.0 mm
Initial Rotation	-0.01 deg
Initial Orientation	Sagittal

Geometry - Navigator

Geometry - Tim Planning Suite

Set-n-Go Protocol	Off
Table position	H
Table position	3 mm
Inline Composing	Off

System - Miscellaneous

Positioning mode	ISO
Table position	H
Table position	3 mm
MSMA	S - C - T
Sagittal	R >> L
Coronal	A >> P
Transversal	F >> H
Coil Combine Mode	Adaptive Combine
Save uncombined	Off
Matrix Optimization	Off
Coil Focus	Flat
AutoAlign	Head > Brain
Coil Select Mode	Default

System - Adjustments

B0 Shim mode	Tune up
Adjust with body coil	Off
Confirm freq. adjustment	Off
Assume Dominant Fat	Off
Assume Silicone	Off
Adjustment Tolerance	Auto

System - Adjust Volume

Position	Isocenter
Orientation	Transversal
Rotation	0.00 deg
A >> P	263 mm
R >> L	350 mm
F >> H	350 mm
Reset	Off

System - Tx/Rx

Frequency 1H	63.685812 MHz
Correction factor	1
Gain	Low
Img. Scale Cor.	1.000
Reset	Off
? Ref. amplitude 1H	0.000 V

Physio - Signal1

1st Signal/Mode	None
TR	1880.0 ms
Concatenations	1

Physio - Cardiac

Magn. preparation	Non-sel. IR
TI	1030 ms
Fat suppr.	None
Dark blood	Off
FoV read	320 mm
FoV phase	100.0 %
Phase resolution	100 %

Physio - PACE

Resp. control	Off
Concatenations	1

Inline - Common

Subtract	On
Save images	On
Autoscaling	Off
Scaling factor	1
Offset	0

Inline - Common

Subtrahend	1
Subtraction group	1
Measurements	1
StdDev	Off
Save original images	On

Inline - MIP

MIP-Sag	Off
MIP-Cor	Off
MIP-Tra	Off
MIP-Time	Off
Save original images	On

Inline - Composing

Inline Composing	Off
Distortion Corr.	On
Mode	2D
Unfiltered images	Off

Inline - MapIt

Save original images	On
MapIt	None
Flip angle	12 deg
Measurements	1
TR	1880.0 ms
TE	2.63 ms

Sequence - Part 1

Introduction	On
Dimension	3D
Elliptical scanning	Off
Reordering	Linear
Asymmetric echo	Allowed
Flow comp.	No
Multi-slice mode	Single shot
Echo spacing	6.6 ms
Bandwidth	200 Hz/Px

Sequence - Part 2

RF pulse type	Fast
Gradient mode	Fast
Excitation	Slab-sel.
RF spoiling	On
Incr. Gradient spoiling	Off
Turbo factor	256

Sequence - Assistant

Mode	Off

\\PHYSIK\E010\Gnirs\BPL_Beekley_Planeboard_Session1_normal\6_20_4PLANE_T2
TA: 9:16 PM: FIX Voxel size: 0.4×0.4×4.0 mmPAT: 2 Rel. SNR: 1.00 : tseR

Properties

Prio recon	Off
Load images to viewer	On
Inline movie	Off
Auto store images	On
Load images to stamp segments	On
Load images to graphic segments	Off
Auto open inline display	Off
Auto close inline display	Off
Start measurement without further preparation	Off
Wait for user to start	Off
Start measurements	Single measurement

Routine

Slice group	1
Slices	80
Dist. factor	0 %
Position	Isocenter
Orientation	Sagittal
Phase enc. dir.	H >> F
AutoAlign	Head > Brain
Phase oversampling	0 %
FoV read	320 mm
FoV phase	100.0 %
Slice thickness	4.0 mm
TR	6450.0 ms
TE	107.0 ms
Averages	3
Concatenations	2
Filter	Distortion Corr.(2D), Prescan Normalize, Elliptical filter
Coil elements	BO1-3;SP3-5

Contrast - Common

TR	6450.0 ms
TE	107.0 ms
TD	0.0 ms
MTC	Off
Magn. preparation	None
Flip angle	180 deg
Fat suppr.	None
Water suppr.	None
Restore magn.	On

Contrast - Dynamic

Averages	3
Averaging mode	Short term
Reconstruction	Magnitude
Measurements	1
Multiple series	Each measurement

Resolution - Common

FoV read	320 mm
FoV phase	100.0 %
Slice thickness	4.0 mm
Base resolution	448
Phase resolution	90 %
Phase partial Fourier	Off
Trajectory	Cartesian
Interpolation	On

Resolution - iPAT

PAT mode	GRAPPA
Accel. factor PE	2
Ref. lines PE	32
Reference scan mode	Self-calibration

Resolution - Filter Image

Image Filter	Off
Distortion Corr.	On
Mode	2D
Unfiltered images	Off
Prescan Normalize	On
Unfiltered images	Off
Normalize	Off
B1 filter	Off

Resolution - Filter Rawdata

Raw filter	Off
Elliptical filter	On

Geometry - Common

Slice group	1
Slices	80
Dist. factor	0 %
Position	Isocenter
Orientation	Sagittal
Phase enc. dir.	H >> F
FoV read	320 mm
FoV phase	100.0 %
Slice thickness	4.0 mm
TR	6450.0 ms
Multi-slice mode	Interleaved
Series	Interleaved
Concatenations	2

Geometry - AutoAlign

Slice group	1
Position	Isocenter
Orientation	Sagittal
Phase enc. dir.	H >> F
AutoAlign	Head > Brain
Initial Position	Isocenter
L	0.0 mm
P	0.0 mm
H	0.0 mm
Initial Rotation	89.99 deg
Initial Orientation	Sagittal

Geometry - Saturation

Fat suppr.	None
Water suppr.	None
Restore magn.	On
Special sat.	None

Geometry - Navigator

Geometry - Tim Planning Suite

Set-n-Go Protocol	Off
Table position	H
Table position	3 mm
Inline Composing	Off

System - Miscellaneous

Positioning mode	FIX
Table position	H
Table position	3 mm
MSMA	S - C - T
Sagittal	R >> L
Coronal	A >> P
Transversal	F >> H
Coil Combine Mode	Adaptive Combine
Save uncombined	Off
Matrix Optimization	Off
Coil Focus	Flat
AutoAlign	Head > Brain
Coil Select Mode	Default

System - Adjustments

B0 Shim mode	Tune up
Adjust with body coil	Off
Confirm freq. adjustment	Off
Assume Dominant Fat	Off
Assume Silicone	Off
Adjustment Tolerance	Auto

System - Adjust Volume

Position	Isocenter
Orientation	Transversal
Rotation	0.00 deg
A >> P	263 mm
R >> L	350 mm
F >> H	350 mm
Reset	Off

System - Tx/Rx

Frequency 1H	63.685812 MHz
Correction factor	1
Gain	High
Img. Scale Cor.	1.000
Reset	Off
? Ref. amplitude 1H	0.000 V

Physio - Signal1

1st Signal/Mode	None
TR	6450.0 ms
Concatenations	2

Physio - Cardiac

Magn. preparation	None
Fat suppr.	None
Dark blood	Off
FoV read	320 mm
FoV phase	100.0 %
Phase resolution	90 %
Trajectory	Cartesian

Physio - PACE

Resp. control	Off
Concatenations	2

Inline - Common

Subtract	Off
Measurements	1
StdDev	Off
Save original images	On

Inline - MIP

MIP-Sag	Off
MIP-Cor	Off
MIP-Tra	Off
MIP-Time	Off
Save original images	On

Inline - Composing

Inline Composing	Off
Distortion Corr.	On
Mode	2D
Unfiltered images	Off

Sequence - Part 1

Introduction	On
Dimension	2D
Compensate T2 decay	Off
Reduce Motion Sens.	On
Contrasts	1
Flow comp.	No
Multi-slice mode	Interleaved
Free echo spacing	Off
Echo spacing	9.74 ms
Bandwidth	199 Hz/Px

Sequence - Part 2

Define	Turbo factor
Echo trains per slice	14
Phase correction	Automatic
Acoustic noise reduction	None
RF pulse type	Normal
Gradient mode	Normal
WARP	Off
Red. EC sensitivity	Off
Turbo factor	15

Sequence - Assistant

Mode	Off
Allowed delay	30 s

\\PHYSIK\E010\Gnirs\BPL_Beekley_Planeboard_Session1_normal\6_20_5PLANE_T1

TA: 8:12 PM: ISO Voxel size: 0.5×0.5×1.0 mmPAT: 2 Rel. SNR: 1.00 : tfl

Properties

Prio recon	Off
Load images to viewer	On
Inline movie	Off
Auto store images	On
Load images to stamp segments	On
Load images to graphic segments	Off
Auto open inline display	Off
Auto close inline display	On
Start measurement without further preparation	Off
Wait for user to start	Off
Start measurements	Single measurement

Routine

Slab group	1
Slabs	1
Dist. factor	50 %
Position	Isocenter
Orientation	Sagittal
Phase enc. dir.	A >> P
AutoAlign	Head > Brain
Phase oversampling	50 %
Slice oversampling	0.0 %
Slices per slab	320
FoV read	320 mm
FoV phase	100.0 %
Slice thickness	1.00 mm
TR	1880.0 ms
TE	2.63 ms
Averages	1
Concatenations	1
Filter	Distortion Corr.(2D), Prescan Normalize, Elliptical filter
Coil elements	BO1-3;SP3-5

Contrast - Common

TR	1880.0 ms
TE	2.63 ms
Magn. preparation	Non-sel. IR
TI	1030 ms
Flip angle	12 deg
Fat suppr.	None
Water suppr.	None

Contrast - Dynamic

Averages	1
Averaging mode	Long term
Reconstruction	Magnitude
Measurements	1
Multiple series	Off

Resolution - Common

FoV read	320 mm
FoV phase	100.0 %
Slice thickness	1.00 mm
Base resolution	320
Phase resolution	100 %
Slice resolution	80 %
Phase partial Fourier	Off
Slice partial Fourier	Off

Resolution - Common

Interpolation	On

Resolution - iPAT

PAT mode	GRAPPA
Accel. factor PE	2
Ref. lines PE	45
Accel. factor 3D	1
Reference scan mode	Integrated

Resolution - Filter Image

Image Filter	Off
Distortion Corr.	On
Mode	2D
Unfiltered images	Off
Prescan Normalize	On
Unfiltered images	Off
Normalize	Off
B1 filter	Off

Resolution - Filter Rawdata

Raw filter	Off
Elliptical filter	On

Geometry - Common

Slab group	1
Slabs	1
Dist. factor	50 %
Position	Isocenter
Orientation	Sagittal
Phase enc. dir.	A >> P
Slice oversampling	0.0 %
Slices per slab	320
FoV read	320 mm
FoV phase	100.0 %
Slice thickness	1.00 mm
TR	1880.0 ms
Multi-slice mode	Single shot
Series	Ascending
Concatenations	1

Geometry - AutoAlign

Slab group	1
Position	Isocenter
Orientation	Sagittal
Phase enc. dir.	A >> P
AutoAlign	Head > Brain
Initial Position	L0.0 P0.0 H3.0
L	0.0 mm
P	0.0 mm
H	3.0 mm
Initial Rotation	-0.01 deg
Initial Orientation	Sagittal

Geometry - Navigator

Geometry - Tim Planning Suite

Set-n-Go Protocol	Off
Table position	H
Table position	3 mm
Inline Composing	Off

System - Miscellaneous

Positioning mode	ISO
Table position	H
Table position	3 mm
MSMA	S - C - T
Sagittal	R >> L
Coronal	A >> P
Transversal	F >> H
Coil Combine Mode	Adaptive Combine
Save uncombined	Off
Matrix Optimization	Off
Coil Focus	Flat
AutoAlign	Head > Brain
Coil Select Mode	Default

System - Adjustments

B0 Shim mode	Tune up
Adjust with body coil	Off
Confirm freq. adjustment	Off
Assume Dominant Fat	Off
Assume Silicone	Off
Adjustment Tolerance	Auto

System - Adjust Volume

Position	Isocenter
Orientation	Transversal
Rotation	0.00 deg
A >> P	263 mm
R >> L	350 mm
F >> H	350 mm
Reset	Off

System - Tx/Rx

Frequency 1H	63.685812 MHz
Correction factor	1
Gain	Low
Img. Scale Cor.	1.000
Reset	Off
? Ref. amplitude 1H	0.000 V

Physio - Signal1

1st Signal/Mode	None
TR	1880.0 ms
Concatenations	1

Physio - Cardiac

Magn. preparation	Non-sel. IR
TI	1030 ms
Fat suppr.	None
Dark blood	Off
FoV read	320 mm
FoV phase	100.0 %
Phase resolution	100 %

Physio - PACE

Resp. control	Off
Concatenations	1

Inline - Common

Subtract	On
Save images	On
Autoscaling	Off
Scaling factor	1
Offset	0

Inline - Common

Subtrahend	1
Subtraction group	1
Measurements	1
StdDev	Off
Save original images	On

Inline - MIP

MIP-Sag	Off
MIP-Cor	Off
MIP-Tra	Off
MIP-Time	Off
Save original images	On

Inline - Composing

Inline Composing	Off
Distortion Corr.	On
Mode	2D
Unfiltered images	Off

Inline - MapIt

Save original images	On
MapIt	None
Flip angle	12 deg
Measurements	1
TR	1880.0 ms
TE	2.63 ms

Sequence - Part 1

Introduction	On
Dimension	3D
Elliptical scanning	Off
Reordering	Linear
Asymmetric echo	Allowed
Flow comp.	No
Multi-slice mode	Single shot
Echo spacing	6.6 ms
Bandwidth	200 Hz/Px

Sequence - Part 2

RF pulse type	Fast
Gradient mode	Fast
Excitation	Slab-sel.
RF spoiling	On
Incr. Gradient spoiling	Off
Turbo factor	256

Sequence - Assistant

Mode	Off

\\PHYSIK\E010\Gnirs\BPL_Beekley_Planeboard_Session1_normal\6_20_5PLANE_T2
TA: 9:16 PM: FIX Voxel size: 0.4×0.4×4.0 mmPAT: 2 Rel. SNR: 1.00 : tseR

Properties

Prio recon	Off
Load images to viewer	On
Inline movie	Off
Auto store images	On
Load images to stamp segments	On
Load images to graphic segments	Off
Auto open inline display	Off
Auto close inline display	Off
Start measurement without further preparation	Off
Wait for user to start	Off
Start measurements	Single measurement

Routine

Slice group	1
Slices	80
Dist. factor	0 %
Position	Isocenter
Orientation	Sagittal
Phase enc. dir.	H >> F
AutoAlign	Head > Brain
Phase oversampling	0 %
FoV read	320 mm
FoV phase	100.0 %
Slice thickness	4.0 mm
TR	6450.0 ms
TE	107.0 ms
Averages	3
Concatenations	2
Filter	Distortion Corr.(2D), Prescan Normalize, Elliptical filter
Coil elements	BO1-3;SP3-5

Contrast - Common

TR	6450.0 ms
TE	107.0 ms
TD	0.0 ms
MTC	Off
Magn. preparation	None
Flip angle	180 deg
Fat suppr.	None
Water suppr.	None
Restore magn.	On

Contrast - Dynamic

Averages	3
Averaging mode	Short term
Reconstruction	Magnitude
Measurements	1
Multiple series	Each measurement

Resolution - Common

FoV read	320 mm
FoV phase	100.0 %
Slice thickness	4.0 mm
Base resolution	448
Phase resolution	90 %
Phase partial Fourier	Off
Trajectory	Cartesian
Interpolation	On

Resolution - iPAT

PAT mode	GRAPPA
Accel. factor PE	2
Ref. lines PE	32
Reference scan mode	Self-calibration

Resolution - Filter Image

Image Filter	Off
Distortion Corr.	On
Mode	2D
Unfiltered images	Off
Prescan Normalize	On
Unfiltered images	Off
Normalize	Off
B1 filter	Off

Resolution - Filter Rawdata

Raw filter	Off
Elliptical filter	On

Geometry - Common

Slice group	1
Slices	80
Dist. factor	0 %
Position	Isocenter
Orientation	Sagittal
Phase enc. dir.	H >> F
FoV read	320 mm
FoV phase	100.0 %
Slice thickness	4.0 mm
TR	6450.0 ms
Multi-slice mode	Interleaved
Series	Interleaved
Concatenations	2

Geometry - AutoAlign

Slice group	1
Position	Isocenter
Orientation	Sagittal
Phase enc. dir.	H >> F
AutoAlign	Head > Brain
Initial Position	Isocenter
L	0.0 mm
P	0.0 mm
H	0.0 mm
Initial Rotation	89.99 deg
Initial Orientation	Sagittal

Geometry - Saturation

Fat suppr.	None
Water suppr.	None
Restore magn.	On
Special sat.	None

Geometry - Navigator

Geometry - Tim Planning Suite

Set-n-Go Protocol	Off
Table position	H
Table position	3 mm
Inline Composing	Off

System - Miscellaneous

Positioning mode	FIX
Table position	H
Table position	3 mm
MSMA	S - C - T
Sagittal	R >> L
Coronal	A >> P
Transversal	F >> H
Coil Combine Mode	Adaptive Combine
Save uncombined	Off
Matrix Optimization	Off
Coil Focus	Flat
AutoAlign	Head > Brain
Coil Select Mode	Default

System - Adjustments

B0 Shim mode	Tune up
Adjust with body coil	Off
Confirm freq. adjustment	Off
Assume Dominant Fat	Off
Assume Silicone	Off
Adjustment Tolerance	Auto

System - Adjust Volume

Position	Isocenter
Orientation	Transversal
Rotation	0.00 deg
A >> P	263 mm
R >> L	350 mm
F >> H	350 mm
Reset	Off

System - Tx/Rx

Frequency 1H	63.685812 MHz
Correction factor	1
Gain	High
Img. Scale Cor.	1.000
Reset	Off
? Ref. amplitude 1H	0.000 V

Physio - Signal1

1st Signal/Mode	None
TR	6450.0 ms
Concatenations	2

Physio - Cardiac

Magn. preparation	None
Fat suppr.	None
Dark blood	Off
FoV read	320 mm
FoV phase	100.0 %
Phase resolution	90 %
Trajectory	Cartesian

Physio - PACE

Resp. control	Off
Concatenations	2

Inline - Common

Subtract	Off
Measurements	1
StdDev	Off
Save original images	On

Inline - MIP

MIP-Sag	Off
MIP-Cor	Off
MIP-Tra	Off
MIP-Time	Off
Save original images	On

Inline - Composing

Inline Composing	Off
Distortion Corr.	On
Mode	2D
Unfiltered images	Off

Sequence - Part 1

Introduction	On
Dimension	2D
Compensate T2 decay	Off
Reduce Motion Sens.	On
Contrasts	1
Flow comp.	No
Multi-slice mode	Interleaved
Free echo spacing	Off
Echo spacing	9.74 ms
Bandwidth	199 Hz/Px

Sequence - Part 2

Define	Turbo factor
Echo trains per slice	14
Phase correction	Automatic
Acoustic noise reduction	None
RF pulse type	Normal
Gradient mode	Normal
WARP	Off
Red. EC sensitivity	Off
Turbo factor	15

Sequence - Assistant

Mode	Off
Allowed delay	30 s

\\PHYSIK\E010\Gnirs\BPL_Beekley_Planeboard_Session1_normal\6_20_6PLANE_T1

TA: 8:12 PM: ISO Voxel size: 1.0×1.0×1.0 mmPAT: 2 Rel. SNR: 1.00 : tfl

Properties

Prio recon	Off
Load images to viewer	On
Inline movie	Off
Auto store images	On
Load images to stamp segments	On
Load images to graphic segments	Off
Auto open inline display	Off
Auto close inline display	On
Start measurement without further preparation	Off
Wait for user to start	Off
Start measurements	Single measurement

Routine

Slab group	1
Slabs	1
Dist. factor	50 %
Position	Isocenter
Orientation	Sagittal
Phase enc. dir.	A >> P
AutoAlign	Head > Brain
Phase oversampling	50 %
Slice oversampling	0.0 %
Slices per slab	320
FoV read	320 mm
FoV phase	100.0 %
Slice thickness	1.00 mm
TR	1880.0 ms
TE	2.63 ms
Averages	1
Concatenations	1
Filter	Distortion Corr.(2D), Prescan Normalize, Elliptical filter
Coil elements	BO1-3;SP3-5

Contrast - Common

TR	1880.0 ms
TE	2.63 ms
Magn. preparation	Non-sel. IR
TI	1030 ms
Flip angle	12 deg
Fat suppr.	None
Water suppr.	None

Contrast - Dynamic

Averages	1
Averaging mode	Long term
Reconstruction	Magnitude
Measurements	1
Multiple series	Off

Resolution - Common

FoV read	320 mm
FoV phase	100.0 %
Slice thickness	1.00 mm
Base resolution	320
Phase resolution	100 %
Slice resolution	80 %
Phase partial Fourier	Off
Slice partial Fourier	Off

Resolution - Common

Interpolation	Off

Resolution - iPAT

PAT mode	GRAPPA
Accel. factor PE	2
Ref. lines PE	45
Accel. factor 3D	1
Reference scan mode	Integrated

Resolution - Filter Image

Image Filter	Off
Distortion Corr.	On
Mode	2D
Unfiltered images	Off
Prescan Normalize	On
Unfiltered images	Off
Normalize	Off
B1 filter	Off

Resolution - Filter Rawdata

Raw filter	Off
Elliptical filter	On

Geometry - Common

Slab group	1
Slabs	1
Dist. factor	50 %
Position	Isocenter
Orientation	Sagittal
Phase enc. dir.	A >> P
Slice oversampling	0.0 %
Slices per slab	320
FoV read	320 mm
FoV phase	100.0 %
Slice thickness	1.00 mm
TR	1880.0 ms
Multi-slice mode	Single shot
Series	Ascending
Concatenations	1

Geometry - AutoAlign

Slab group	1
Position	Isocenter
Orientation	Sagittal
Phase enc. dir.	A >> P
AutoAlign	Head > Brain
Initial Position	L0.0 P0.0 H3.0
L	0.0 mm
P	0.0 mm
H	3.0 mm
Initial Rotation	-0.01 deg
Initial Orientation	Sagittal

Geometry - Navigator

Geometry - Tim Planning Suite

Set-n-Go Protocol	Off
Table position	H
Table position	3 mm
Inline Composing	Off

System - Miscellaneous

Positioning mode	ISO
Table position	H
Table position	3 mm
MSMA	S - C - T
Sagittal	R >> L
Coronal	A >> P
Transversal	F >> H
Coil Combine Mode	Adaptive Combine
Save uncombined	Off
Matrix Optimization	Off
Coil Focus	Flat
AutoAlign	Head > Brain
Coil Select Mode	Default

System - Adjustments

B0 Shim mode	Tune up
Adjust with body coil	Off
Confirm freq. adjustment	Off
Assume Dominant Fat	Off
Assume Silicone	Off
Adjustment Tolerance	Auto

System - Adjust Volume

Position	Isocenter
Orientation	Transversal
Rotation	0.00 deg
A >> P	263 mm
R >> L	350 mm
F >> H	350 mm
Reset	Off

System - Tx/Rx

Frequency 1H	63.685812 MHz
Correction factor	1
Gain	Low
Img. Scale Cor.	1.000
Reset	Off
? Ref. amplitude 1H	0.000 V

Physio - Signal1

1st Signal/Mode	None
TR	1880.0 ms
Concatenations	1

Physio - Cardiac

Magn. preparation	Non-sel. IR
TI	1030 ms
Fat suppr.	None
Dark blood	Off
FoV read	320 mm
FoV phase	100.0 %
Phase resolution	100 %

Physio - PACE

Resp. control	Off
Concatenations	1

Inline - Common

Subtract	On
Save images	On
Autoscaling	Off
Scaling factor	1
Offset	0

Inline - Common

Subtrahend	1
Subtraction group	1
Measurements	1
StdDev	Off
Save original images	On

Inline - MIP

MIP-Sag	Off
MIP-Cor	Off
MIP-Tra	Off
MIP-Time	Off
Save original images	On

Inline - Composing

Inline Composing	Off
Distortion Corr.	On
Mode	2D
Unfiltered images	Off

Inline - MapIt

Save original images	On
MapIt	None
Flip angle	12 deg
Measurements	1
TR	1880.0 ms
TE	2.63 ms

Sequence - Part 1

Introduction	On
Dimension	3D
Elliptical scanning	Off
Reordering	Linear
Asymmetric echo	Allowed
Flow comp.	No
Multi-slice mode	Single shot
Echo spacing	6.6 ms
Bandwidth	200 Hz/Px

Sequence - Part 2

RF pulse type	Fast
Gradient mode	Fast
Excitation	Slab-sel.
RF spoiling	On
Incr. Gradient spoiling	Off
Turbo factor	256

Sequence - Assistant

Mode	Off

\\PHYSIK\E010\Gnirs\BPL_Beekley_Planeboard_Session1_normal\6_20_6PLANE_T2
TA: 9:16 PM: FIX Voxel size: 0.4×0.4×4.0 mmPAT: 2 Rel. SNR: 1.00 : tseR

Properties

Prio recon	Off
Load images to viewer	On
Inline movie	Off
Auto store images	On
Load images to stamp segments	On
Load images to graphic segments	Off
Auto open inline display	Off
Auto close inline display	Off
Start measurement without further preparation	Off
Wait for user to start	Off
Start measurements	Single measurement

Routine

Slice group	1
Slices	80
Dist. factor	0 %
Position	Isocenter
Orientation	Sagittal
Phase enc. dir.	H >> F
AutoAlign	Head > Brain
Phase oversampling	0 %
FoV read	320 mm
FoV phase	100.0 %
Slice thickness	4.0 mm
TR	6450.0 ms
TE	107.0 ms
Averages	3
Concatenations	2
Filter	Distortion Corr.(2D), Prescan Normalize, Elliptical filter
Coil elements	BO1-3;SP3-5

Contrast - Common

TR	6450.0 ms
TE	107.0 ms
TD	0.0 ms
MTC	Off
Magn. preparation	None
Flip angle	180 deg
Fat suppr.	None
Water suppr.	None
Restore magn.	On

Contrast - Dynamic

Averages	3
Averaging mode	Short term
Reconstruction	Magnitude
Measurements	1
Multiple series	Each measurement

Resolution - Common

FoV read	320 mm
FoV phase	100.0 %
Slice thickness	4.0 mm
Base resolution	448
Phase resolution	90 %
Phase partial Fourier	Off
Trajectory	Cartesian
Interpolation	On

Resolution - iPAT

PAT mode	GRAPPA
Accel. factor PE	2
Ref. lines PE	32
Reference scan mode	Self-calibration

Resolution - Filter Image

Image Filter	Off
Distortion Corr.	On
Mode	2D
Unfiltered images	Off
Prescan Normalize	On
Unfiltered images	Off
Normalize	Off
B1 filter	Off

Resolution - Filter Rawdata

Raw filter	Off
Elliptical filter	On

Geometry - Common

Slice group	1
Slices	80
Dist. factor	0 %
Position	Isocenter
Orientation	Sagittal
Phase enc. dir.	H >> F
FoV read	320 mm
FoV phase	100.0 %
Slice thickness	4.0 mm
TR	6450.0 ms
Multi-slice mode	Interleaved
Series	Interleaved
Concatenations	2

Geometry - AutoAlign

Slice group	1
Position	Isocenter
Orientation	Sagittal
Phase enc. dir.	H >> F
AutoAlign	Head > Brain
Initial Position	Isocenter
L	0.0 mm
P	0.0 mm
H	0.0 mm
Initial Rotation	89.99 deg
Initial Orientation	Sagittal

Geometry - Saturation

Fat suppr.	None
Water suppr.	None
Restore magn.	On
Special sat.	None

Geometry - Navigator

Geometry - Tim Planning Suite

Set-n-Go Protocol	Off
Table position	H
Table position	3 mm
Inline Composing	Off

System - Miscellaneous

Positioning mode	FIX
Table position	H
Table position	3 mm
MSMA	S - C - T
Sagittal	R >> L
Coronal	A >> P
Transversal	F >> H
Coil Combine Mode	Adaptive Combine
Save uncombined	Off
Matrix Optimization	Off
Coil Focus	Flat
AutoAlign	Head > Brain
Coil Select Mode	Default

System - Adjustments

B0 Shim mode	Tune up
Adjust with body coil	Off
Confirm freq. adjustment	Off
Assume Dominant Fat	Off
Assume Silicone	Off
Adjustment Tolerance	Auto

System - Adjust Volume

Position	Isocenter
Orientation	Transversal
Rotation	0.00 deg
A >> P	263 mm
R >> L	350 mm
F >> H	350 mm
Reset	Off

System - Tx/Rx

Frequency 1H	63.685812 MHz
Correction factor	1
Gain	High
Img. Scale Cor.	1.000
Reset	Off
? Ref. amplitude 1H	0.000 V

Physio - Signal1

1st Signal/Mode	None
TR	6450.0 ms
Concatenations	2

Physio - Cardiac

Magn. preparation	None
Fat suppr.	None
Dark blood	Off
FoV read	320 mm
FoV phase	100.0 %
Phase resolution	90 %
Trajectory	Cartesian

Physio - PACE

Resp. control	Off
Concatenations	2

Inline - Common

Subtract	Off
Measurements	1
StdDev	Off
Save original images	On

Inline - MIP

MIP-Sag	Off
MIP-Cor	Off
MIP-Tra	Off
MIP-Time	Off
Save original images	On

Inline - Composing

Inline Composing	Off
Distortion Corr.	On
Mode	2D
Unfiltered images	Off

Sequence - Part 1

Introduction	On
Dimension	2D
Compensate T2 decay	Off
Reduce Motion Sens.	On
Contrasts	1
Flow comp.	No
Multi-slice mode	Interleaved
Free echo spacing	Off
Echo spacing	9.74 ms
Bandwidth	199 Hz/Px

Sequence - Part 2

Define	Turbo factor
Echo trains per slice	14
Phase correction	Automatic
Acoustic noise reduction	None
RF pulse type	Normal
Gradient mode	Normal
WARP	Off
Red. EC sensitivity	Off
Turbo factor	15

Sequence - Assistant

Mode	Off
Allowed delay	30 s
